# Genome-wide identification and characterization of SPL transcription factor family and their evolution and expression profiling analysis in cotton

**DOI:** 10.1038/s41598-017-18673-4

**Published:** 2018-01-15

**Authors:** Caiping Cai, Wangzhen Guo, Baohong Zhang

**Affiliations:** 1 State Key Laboratory of Crop Genetics & Germplasm Enhancement, Hybrid Cotton R&D Engineering Research Center, Ministry of Education, Nanjing Agricultural University, Nanjing, 210095 China; 2 Department of Biology, East Carolina University, Greenville, NC 27858 USA

## Abstract

Plant specific transcription factors, SQUAMOSA promoter-binding protein-like (SPL), are involved in many biological processes. However, no systematical study has been reported in cotton. In this study, a total of 177 *SPL* genes were identified, including 29, 30, 59 and 59 *SPLs* in *Gossypium arboreum*, *G. raimondii*, *G. barbadense*, and *G. hirsutum*, respectively. These *SPL* genes were classified into eight phylogenetical groups. The gene structure, conserved motif, and clustering were highly conserved within each orthologs. Two zinc finger-like structures (Cys3His and Cys2HisCys) and NLS segments were existed in all GrSPLs. Segmental duplications play important roles in SPL family expansion, with 20 genes involved in segmental duplications and 2 in tandem duplications, and ten ortholog pairs in syntenic regions between *G. raimondii* and *A. thaliana*. Several putative cis-elements, involved in light, stresses and phytohormones response, were found in the promoter regions of *GhSPLs*, suggesting that plant responses to those environmental changes may be induced through targeting SPL transcription factors. RNA-seq analysis shows that SPL genes were differentially expressed in cotton; some were highly expressed during fiber initiation and early development. Comparing with other plants, SPL genes show subfunctionalization, lost and/or gain functions in cotton during long-term domestication and evolution.

## Introduction

SQUAMOSA promoter-binding protein-like (SPL), one class of plant-specific transcription factors, have a highly conserved SBP domains (for SQUAMOSA-PROMOTER BINDING PROTEIN) with approximately 78 amino acids in length, and containing an eight Cys or His sequence motif (two Zn-finger like structure) and contained a nuclear localization signal (NLS) motif. The two Zn-finger binding sites consist a Cys3HisCys2HisCys or Cys6HisCys, in which the first zinc finger is Cys3His or Cys4, and second is Cys2HisCys^1^. SBP1 and SBP2 are the first two members of the SBP/SPL gene family, which were identified in *Antirrhinum majus* floral meristem involved in the control of early flower development^2^. Many SPL members were targeted by the miR156/157, and the miR156/SPL module plays important roles in diverse developmental processes in *Arabidopsis*, including shoot development, the phase change from vegetative growth to reproductive growth, and tolerance to abiotic stresses^3–5^. Various functions of *SPL* genes were also reported in other plant species, including governing yield-related traits in hexaploid wheat^6^, redundantly initiating side tillers and affecting biomass yield of energy crop in switchgrass^7^, regulating floral organ size and ovule production in cotton^8^, and regulating ovary and fruit development in tomato^9^.

Genome-wide identification of SPL/SBP-box gene family have been characterized in many plant species, including potato^10^, soybean^11^, oilseed rape^12^, pepper^13^, peanut^14^, Chinese cabbage^15^, citrus^16^, *Prunus mume*^17^, *Salvia miltiorrhiza* (Danshen)^18^, castor bean^19^, *Populus trichocarpa*^20^, apple^21^, grape^22^, tomato^23^, *Arabidopsis* and rice^24^. Guo *et al*.^25^ reported identification and phylogenetic relationship of 120 SPL genes from nine species representing the main green plant lineages: green alga, moss, lycophyte, gymnosperm and angiosperm. However, the identification and functional analysis of SPL gene family is much beyond in cotton than that in other plant species. There is only one report in which Zhang and colleagues^26^ cloned 24 *SPL* genes in cotton.

Cotton (*Gossypium* spp.) is one of the most important economic crops, and provides natural textile fiber and oilseed. *G. hirsutum* L. (AD1) and *G. barbadense* L. (AD2) are two tetraploids cultivated species, which were formed about 1–2 million years ago (MYA) through interspecific hybridization and chromosome doubling of A-genome (resembling A2 *G. arboreum*) and D-genomes (resembling D5 *G. raimondii*)^27,28^. Recently, the whole-genome sequences of four cotton species were released, including two allotetraploid species *G. hirsutum* acc. TM-1^29^ and *G. barbadense*, cv. Xinhai21 and acc. 3–79^30,31^ and their two diploid progenitors *G. arboreum*^32^ and *G. raimondii*^33,34^. Those genome sequences provide a possible to identify *SPL* genes at a genome-wide level in cotton.

In this study, we identified *SPL* genes in four cotton species, defined the corresponding relationships and chromosomal locations. We built a phylogenetic tree of the SPL gene family in *Gossypium*, *A. thaliana*, *O. sativa* and *P. trichocarpa*, and carried out a genome-wide intra- and inter-genomic duplication analysis of *G. raimondii* and other three plant species. Additionally, we systematically analyzed the gene structure, conserved motif, cis-acting elements and expression pattern of all identified *GhSPL* genes in *G. hirsutum*. The results will provide a solid foundation to understand the distribution, structure and evolution of the SPL gene family in cotton, and will contribute to investigate of the detailed functional differentiation and application of these genes in the future.

## Results

### Genome-wide identification of the SPL gene family in *Gossypium* and their chromosomal distribution

To identify the SQUAMOSA promoter binding protein-like (SPL or SBP) transcription factor genes in cotton, SBP domain (PF03110) was used to search protein database of four cotton species, *G. raimondii*^33^, *G. arboreum*^32^, *G. hirsutum* acc. TM-1^29^, and *G. barbadense* acc. 3–79^31^ by HMMER^35^. The candidate *SPL* genes were verified by the presence of the SBP domain using SMART and CDD^36,37^. A total of 177 *SPL* genes were identified in cotton, including 29, 30, 59 and 59 were identified in *G. arboretum*, *G. raimondii*, *G. hirsutum* and *G. barbadense*, respectively (Table S1). *GrSPL* genes were named according to the closest orthologs in *A. thaliana*. Among 17 SPL genes in *A. thaliana*, *AtSPL3*, *AtSPL4*, *AtSPL11*, *AtSPL12*, *AtSPL1*5 and *AtSPL16* have not orthologs in *G. raimondii*; and all 30 *GrSPLs* from the rest of 10 *AtSPLs* orthologs. *AtSPL13* has 5 paralogs in *G. raimondii*; *AtSPL1*, *AtSPL5* and *AtSPL6* have 4 paralogs; *AtSPL2*, and *AtSPL9* have 3 paralogs; *AtSPL7*, *AtSPL8* and *AtSPL10* have 2 paralogs; and *AtSPL14* only 1 paralogs in *G. raimondii*. Different paralogs were coded a, b, c, and so on, according to their order of the homologous chromosomes. The corresponding orthologs in *G. arboreum*, *G. hirsutum* acc. TM-1 and *G. barbadense* acc. 3–79 were named as *GaSPL*, *GhSPL*, and *GbSPL*, respectively. The encoded protein lengths of 59 SPL genes varied from 141 to 1083 amino acids, and the predicted molecular weight (Mw) of these SPL proteins ranged from 16.112 to 119.882 kDa in upland cotton *G. hirsutum* (Table S1).

**Figure 1:**
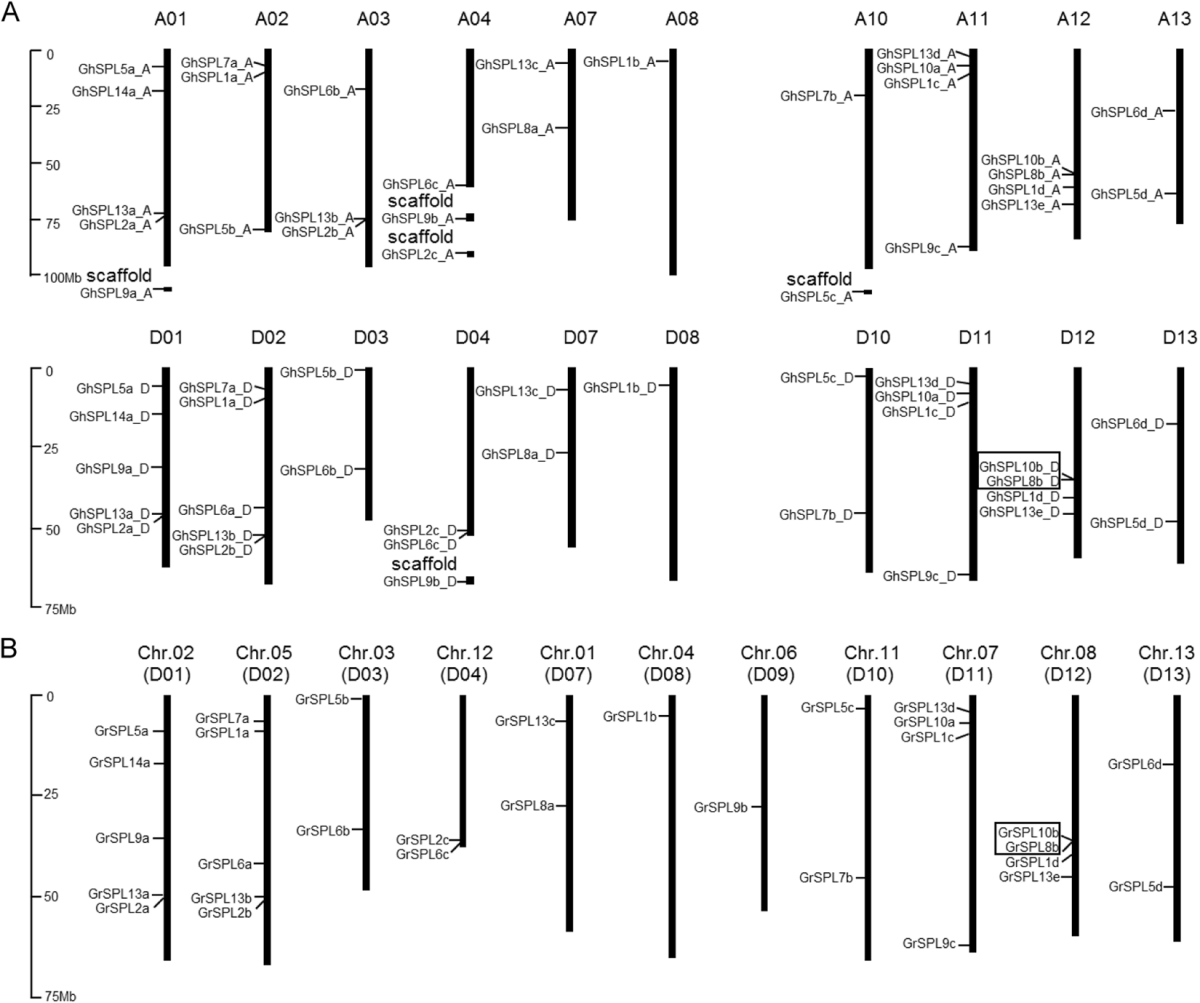
Chromosomal distribution of *SPL* genes in *Gossypium*. The putative *SPL* genes of *G. hirsutum* and *G. raimondii* were shown on (**A**) and (**B**), respectively. The scale represents megabases (Mb). Tandem duplicated genes were marked with black outlined boxes.

The 59 *GhSPL* genes were located on 20 chromosomes, with no *SPL* gene detected in A5/D5, A6/D6, and A9/D9 homologous chromosomes in *G. hirsutum* acc. TM-1 (Fig. 1, Table S1). 30 *GrSPL* genes were located on 11 chromosomes in *G. raimondii* except on the D5 and D6. *GrSPL9b* was located on the D9 in *G. raimondii*, but *GhSPL9b_A*/*GhSPL9b_D* were in A4/D4 (scaffold) homologous chromosomes in *G. hirsutum*. This phenomenon, *SPL9b* was positioned on the different chromosomes, might be caused by assembly error in the duplicated gene regions, and need to be further confirmed. Furthermore, tandem duplication events were defined as genes separated by five or fewer genes and within 100 Kb region^38^. Only one gene pairs on D12 (*GrSPL10b*/*GrSPL8b*) displayed tandem duplications in *G. raimondii* (Fig. 1B), and the corresponding tandem duplication (*GhSPL10b_D*/*GhSPL8b_D*) was detected on D12 of *G. hirsutum* (Fig. 1A). On A12, the distance was over 100 Kb (161.95 Kb) between *GhSPL10b_A* and *GhSPL8b_A*. Compared with the distribution of *SPL* genes in *G. raimondii* and *G. hirsutum*, *SPLs* displayed high collinearity in the D-genome of *G. raimondii* and A-, D-subgenomes of *G. hirsutum*. Additionally, two pairs of orthologs genes were located in A2/A3 reciprocal translocation section, within *GhSPL13b_A and GhSPL2b_A* on A3 chromosomes, *GhSPL13b_D and GhSPL2b_D* on D2.

### Phylogenetic analysis and gene duplication observation of SPL gene family

**Figure 2:**
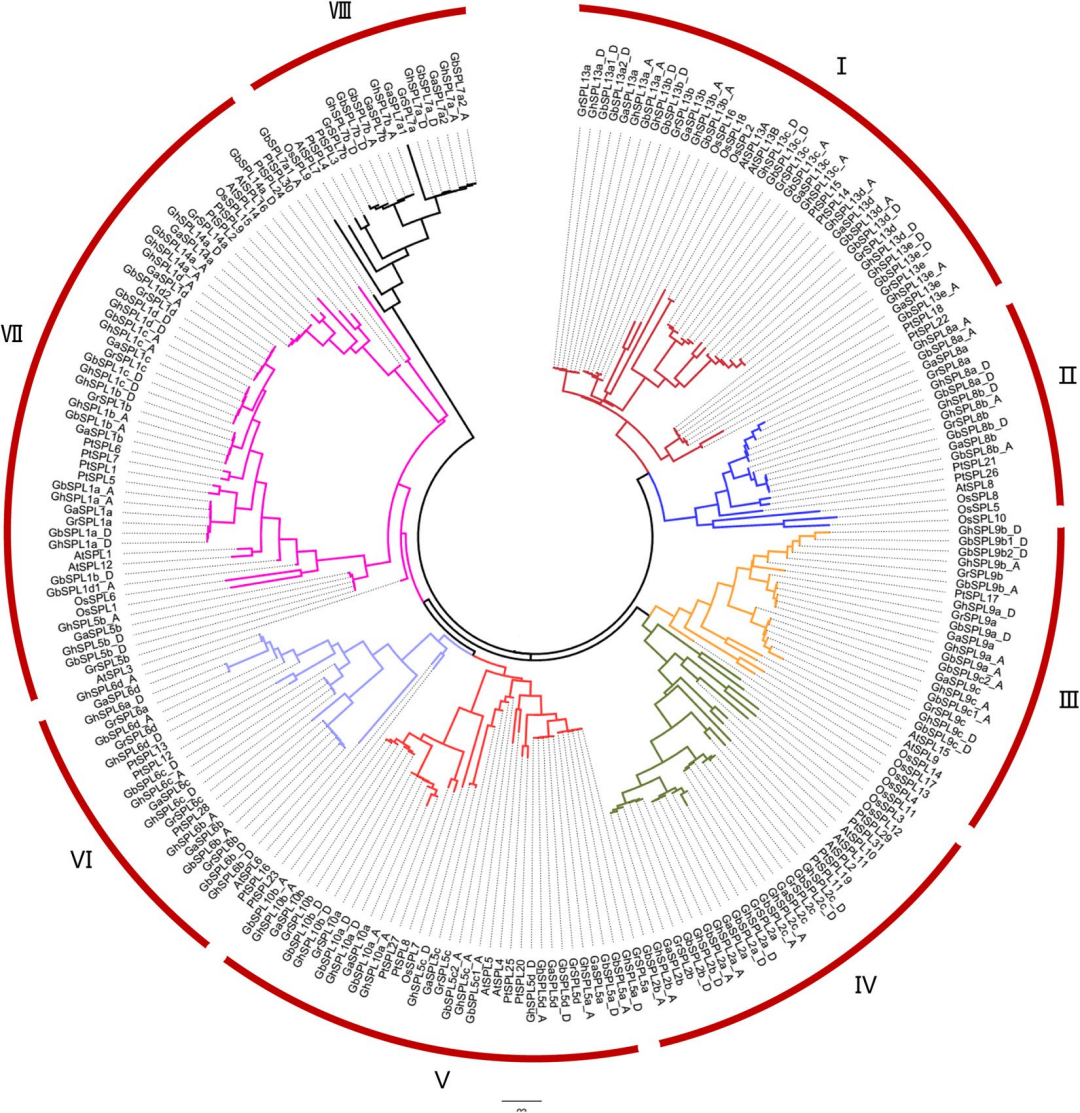
Phylogenetic relationships of SPL proteins from cotton species (*G. arboreum*, *G. raimondii*, *G. hirsutum* acc. TM-1, and *G. barbadense* acc. 3–79), *A. thaliana*, *O. sativa*, and *P. trichocarpa*. The unrooted NJ tree was constructed using MEGA 7, and the bootstrap test was performed with 1000 replicates.

To investigate the phylogenetic relationship of the SPL transcription factor family in cotton, a total of 242 SPLs were used to construct a Neighbour-Joining (N-J) phylogenetic tree by MEGA 7.0 software^39^. These 242 SPLs included 177 obtained from this research in four cotton species, *G. raimondii* (30), *G. arboretum* (29), *G. hirsutum* (59) and *G. barbadense* (59); the rest 65 were obtained from three well-studied plant species, including 17 from *A. thaliana*, 18 from *O. sativa* and 30 from *P. trichocarpa*. As shown in Fig. 2, all of the SPL genes were clustered into eight sub-groups (from I to VIII), and each group contained at least one protein from three species (*Arabidopsis*, rice and poplar) except group VI in which there was no SPL from rice. Additionally, different SPL orthologs were clearly distinguished in cotton. Cotton SPL13, SPL8, SPL9, SPL2, SPL6 and SPL7 were grouped to cluster I–IV, VI and VIII, respectively. Cotton SPL5 and SPL10 were clustered in group V, SPL1 and SPL14 were clustered in group VII.

**Figure 3:**
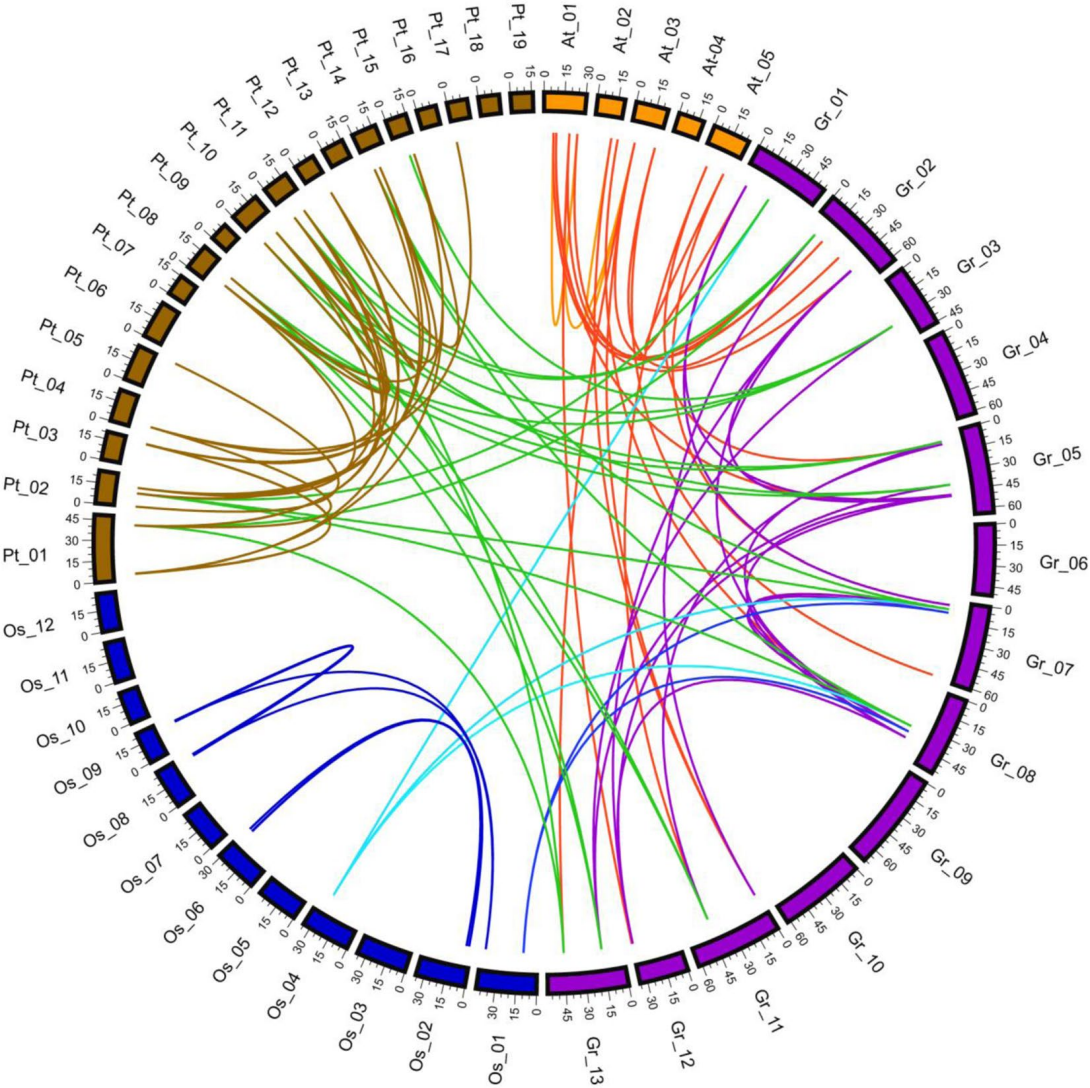
Distribution of the intra- and inter-genomic duplications of SPL genes in *G. raimondii*, *A. thaliana*, *O. sativa* and *P. trichocarpa. G. raimondii*, *A. thaliana*, *O. sativa* and *P. trichocarpa* chromosomes made a circle using CIRCOS. The different species chromosomes and their intra-genomic duplication were depicted with different colors. Intra-genomic duplication between *G. raimondii* and other three species (*A. thaliana*, *O. sativa* and *P. trichocarpa*) were indicated with red, cyan and forestgreen lines, respectively.

To reveal *SPL* duplication events, four whole intra- genomic duplication data files of *G. raimondii*, *A. thaliana*, *O. sativa* and *P. trichocarpa*, and three inter-genomic duplication data files between *G. raimondii* and three other species were downloaded from the PGDD database^40^. All *SPL* gene duplication events was filtered out (Fig. 3, Table S2). Among 30 *SPL* genes in *G. raimondii*, we identified 16 pairs of duplications out of 20 *GrSPLs* (*GrSPL1a*/*GrSPL1d*, *GrSPL1c*/*GrSPL1d*, *GrSPL2a*/*GrSPL2b*, *GrSPL2a*/*GrSPL2c*, *GrSPL2b*/*GrSPL2c*, *GrSPL5a*/*GrSPL5c*, *GrSPL6a*/*GrSPL6d*, *GrSPL6b*/*GrSPL6d*, *GrSPL7a*/*GrSPL7b*, *GrSPL10a*/*GrSPL10b*, *GrSPL13a*/*GrSPL13b*, *GrSPL13a*/*GrSPL13e*, *GrSPL13b*/*GrSPL13e*, *GrSPL13c*/*GrSPL13b*, *GrSPL13c*/*GrSPL13d* and *GrSPL13e*/*GrSPL2c*), involving 7 *GrSPL* orthologs except *GrSPL8*, *GrSPL9* and *GrSPL14*. All duplication pairs had Ka/Ks values of less than 1 (ranging 0.16–0.50) (Table S2), suggesting that SNP gene family in *G. raimondii* had subjected to purifying selection during the long-term evolutionary process. Compared with tandem duplications (only one pair on D12, *GrSPL10b*/*GrSPL8b*), segmental duplications played a significant role in expansion of *SPL* gene family in cotton. As well as, 4, 7 and 25 pairs of duplications were identified in *A. thaliana*, *O. sativa* and *P. trichocarpa*, respectively (Fig. 3, Table S2).

*SPL* duplication pattern between *G. raimondii* and three other species, *A. thaliana*, *O. sativa* and *P. trichocarpa* were also analyzed. Among 30 *GrSPLs* from 10 *Arabidopsis* SPL orthologs, 19 pairs of duplication events were identified between *G. raimondii* and *A. thaliana*, involving 8 *GrSPL* orthologs, and 14 *GrSPL and 12 AtSPL genes*, respectively; 22 pairs of duplication events were identified between *G. raimondii* and *P. trichocarpa*, involving 5 *GrSPL* orthologs, and 10 *GrSPL and 11 PtSPL genes*, respectively; only 5 pairs of duplications were observed in *G. raimondii* and *O. sativa*, and this indicated that SPL gene family of *Gossypium* and *O. sativa* were less conserved. There were more *GrSPL* genes or orthologs between *G. raimondii* and *A. thaliana* than that between *G. raimondii* and *P. trichocarpa*. We compared syntenic map of *G. raimondii* and *A. thaliana* (Fig. 3, Table S2), with ten ortholog pairs positions on segmental duplicated blocks including the following: *GrSPL1d*/*AtSPL1*; *GrSPL2a*, *2b*/*AtSPL11*; *GrSPL5a*/*AtSPL4*, *5*; *GrSPL5c*/*AtSPL3*, 5; *GrSPL5d*/*AtSPL5*; *GrSPL6c*/*AtSPL6*; *GrSPL7a*, *7b*/*AtSPL7*; *GrSPL9a*, *9c*/*AtSPL9*, 15; *GrSPL13c*, *13d*/*AtSPL13B*; and *GrSPL14a*/*AtSPL14*, 16.

### Gene structure and conserved motif analysis of SPLs in *G. hirsutum*

**Figure 4:**
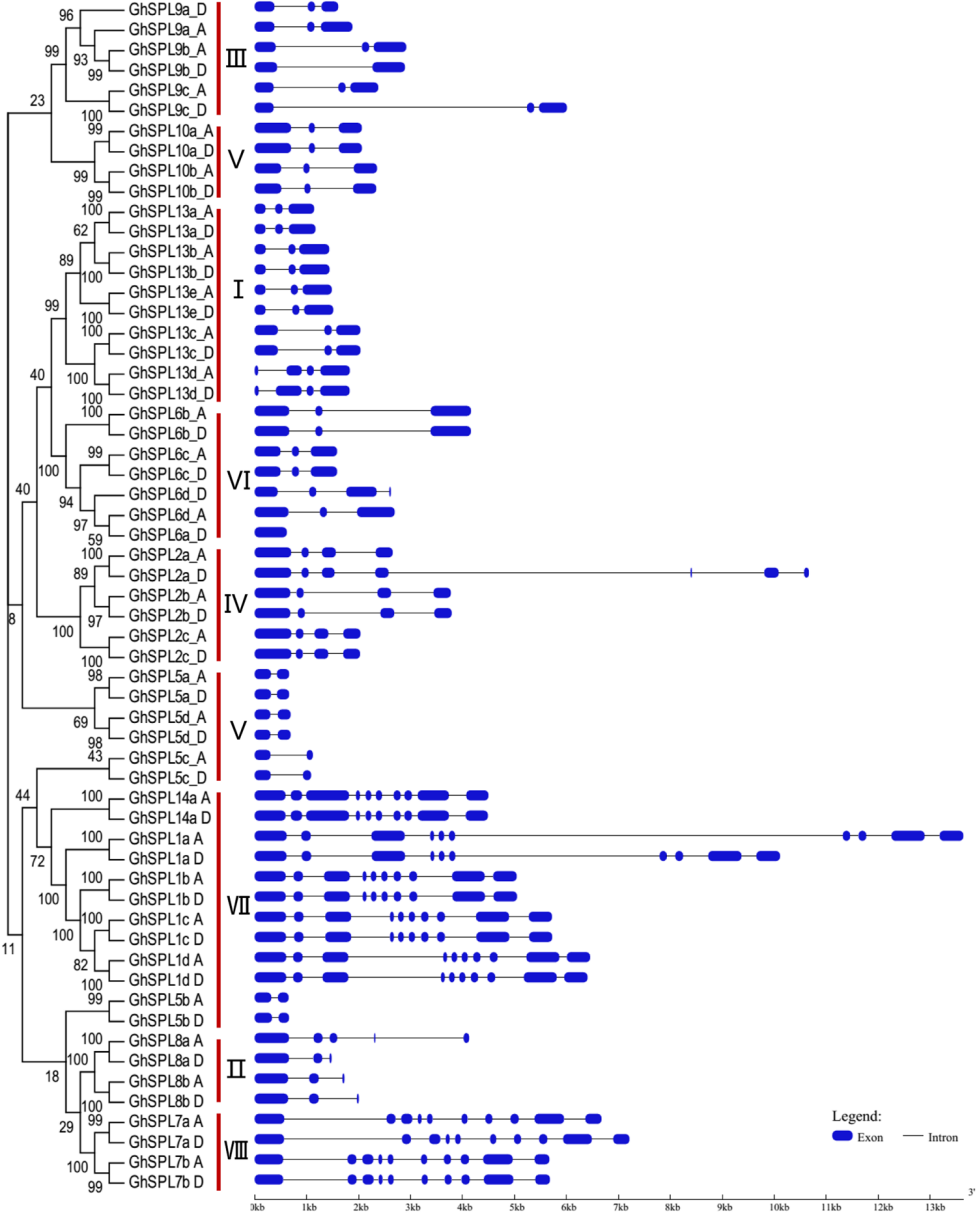
Phylogenetic analysis and gene structure of *GhSPL*s. (**A**) The unrooted NJ tree of 59 *GhSPL* genes was constructed using MEGA 7, and the bootstrap test was performed with 1000 replicates. (**B**) The exon/intron distribution of *GhSPL* genes. Exons and introns were represented by black boxes and lines, respectively.

With *GhSPLs* as an example, we analyzed the *SPL* gene exon/intron structure, conserved motif, and putative cis-acting elements from *GhSPLs* promoters. An unrooted N-J tree was also constructed only using 59 SPL protein sequences from *G. hirsutum* (Fig. 4A). The gene structures of 59 *GhSPL*s were analyzed by GSDS 2.0^41^, and displayed in Fig. 4B. The number of introns of 59 *GhSPLs* varied from 0 to 9. Nearly half of *SPL* genes (27 *GhSPLs*) had two introns (6 *GhSPL6*, 3 *GhSPL8*, 6 *GhSPL9*, 4 *GhSPL10* and 8 *GhSPL13*); 5 *GhSPL*2 and 8 *GhSPL5* had three and one introns, respectively; 14 *GhSPLs* had nine introns (including 8 *GhSPL1*, 4 *GhSPL7* and 2 *GhSPL14*); the remaining SPL members *GhSPL2a_D*, *GhSPL8a_A*, *GhSPL13d_A*/*D* and *GhSPL6a_D* had 6, 4, 3 and 0 introns, respectively. By comparing the SPL gene structures of *G. hirsutum* and *Arabidopsis*, we found that the pattern of exon/intron structures of *SPLs* in *G. hirsutum* is quite similar to *AtSPLs*. In *Arabidopsis*, *AtSPL1*, *AtSPL7* and *AtSPL14* had 9 introns; *AtSPL6*, *AtSPL8 AtSPL9*, *AtSPL10* and *AtSPL13* had 2 introns; *AtSPL2* and *AtSPL5* had 3 and 1 introns, respectively (supplementary material Fig. S1). This result reveals that different *GhSPL* orthologs exhibited different exon-intron structures and were similar to *Arabidopsis* orthologs.

**Figure 5:**
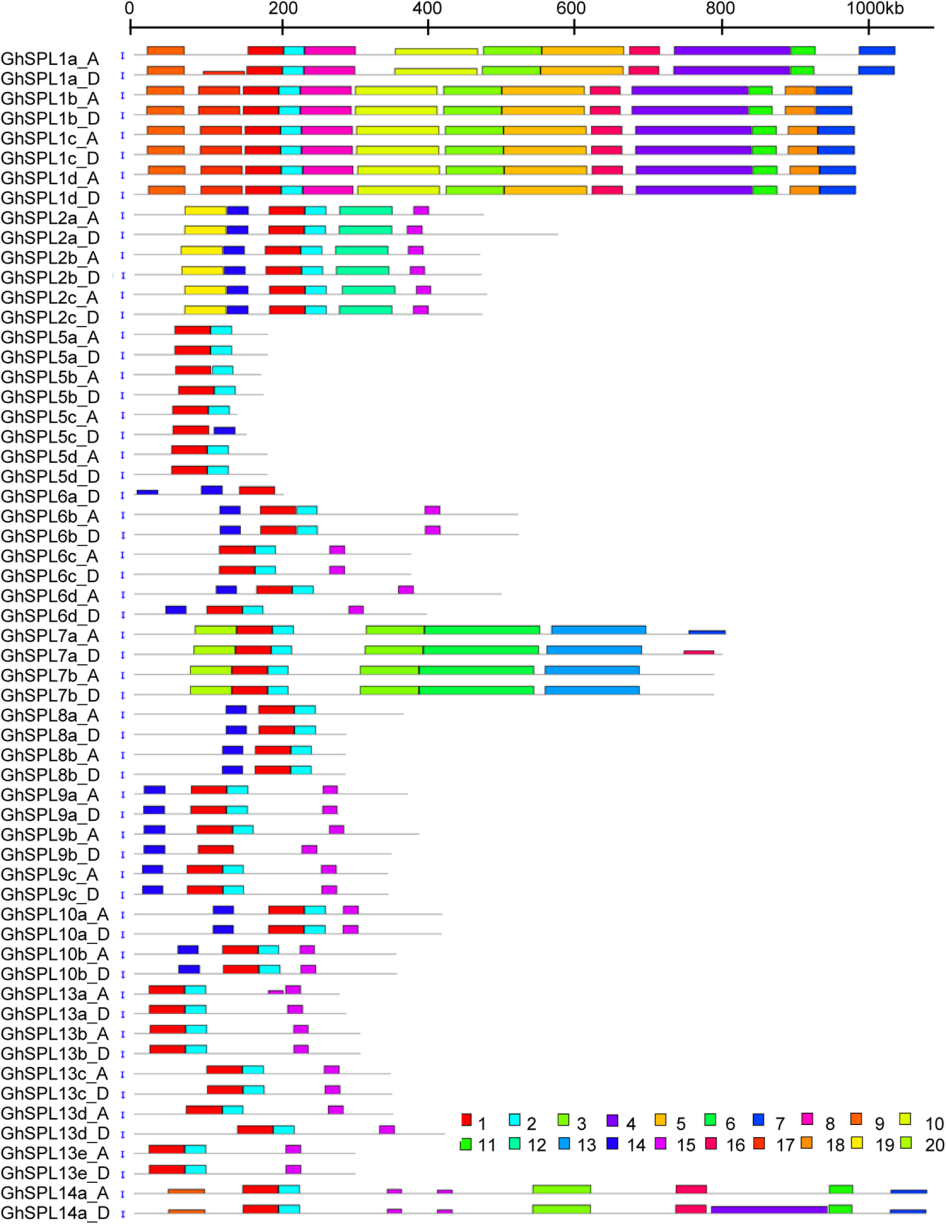
Conserved motifs of GhSPL proteins. Based on the GhSPL protein sequences, the conserved motifs were identified using MEME (suite 4.11.4), and each motif is indicated with a colored box numbered (1 to 20) at the bottom. Motif 1 and motif 2 were two Zn-finger like structure and nuclear localization signal (NLS). Motif 7 contained the miR156/miR157 recognition element as a target site for the miR156/miR157 in 3′ UTR.

**Figure 6:**
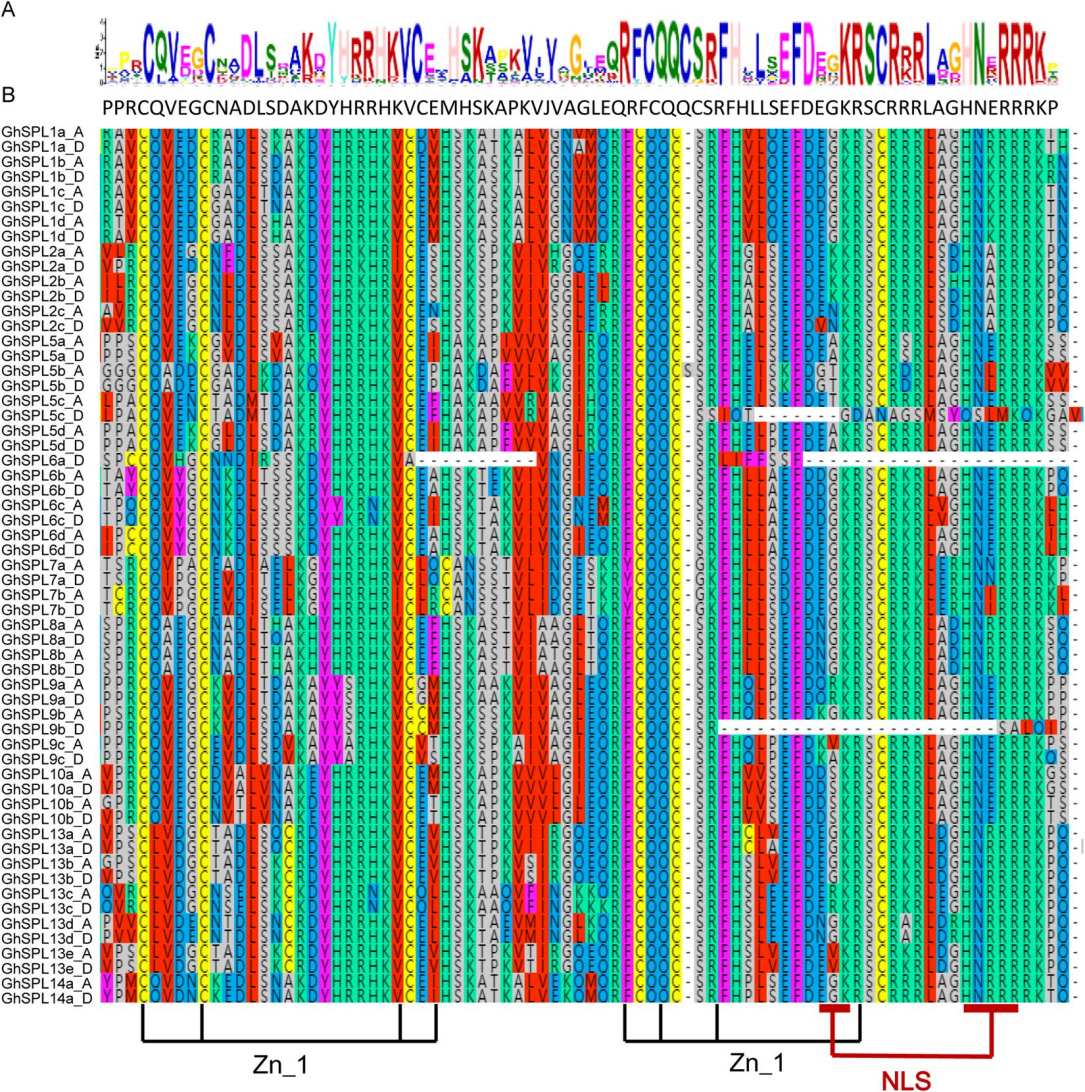
Alignment of the SBP domain in GhSPL proteins. (**A**) Motif logo and protein sequence of the SBP domain and NLS segment. (**B**) Multiple sequences alignment was performed using MAFFT version 7. Two Zn-finger like structure (Cys3His-type, Cys2HisCys) and NLS are indicated.

The MEME^42^ was used to predict motifs of 59 SPL protein sequences in *G. hirsutum*. 20 motifs, named motifs 1 to 20 were identified (Fig. 5). The length of 20 identified motifs and consensus sequence were listed in supplementary material Table S3, and Logos of 20 conserved motifs are shown in supplementary material Fig. S2. The lengths of those conserved motifs were between 21 (motif 15) and 159 amino acids (motif 4). The number of the conserved motifs in each *GhSPL* protein varied from 2 to 13. All GhSPL proteins contained motif 1 (two Zn-finger like structure), and 56 *GhSPL*s contained motif 2 (nuclear localization signal, NLS) except GhSPL5c_D, GhSPL6a_D and GhSPL9b_D. Seven GhSPL5 proteins only had motifs 1 and 2; GhSPL8 had motifs 1, 2 and 14; GhSPL13 had motifs 1, 2 and 15; GhSPL9 and GhSPL10 had motifs 1, 2 14, and 15; other GhSPLs had more motifs, such as GhSPL2 (6 motifs), GhSPL7 (6–7 motifs), GhSPL14 (9–10 motifs) and GhSPL1 (11–13 motifs). Then, multiple alignment of all 59 GhSPL proteins was performed by MAFFT version 7, and presented the SBP domain structures in detail. All GhSPLs exhibit two zinc finger-like structures and NLS segments, with the exception of three SPLs (GhSPL5c_D, GhSPL6a_D and GhSPL9b_D) which lacked NLS. The first Zn-finger like structure (Cys3His), the second Zn-finger like structure (Cys2HisCys) and highly conserved NLS were signed in Fig. 6B. The SBP domain motif logo and protein sequence were showed in Fig. 6A. Therefore, the sequences SBP domain of *GhSPLs*, two Zn-finger structure and NLS section, were also conserved in cotton, and SPL motif member architecture within each of *GhSPL* orthologs tend to have a similar number and type of motifs.

In conclusion, members belonging to the same *GhSPL* orthologs had a similar gene structure, motif architecture, tended to cluster together in phylogenetic tree.

### The cis-acting elements analysis of *GhSPL* gene promoter regions

**Figure 7:**
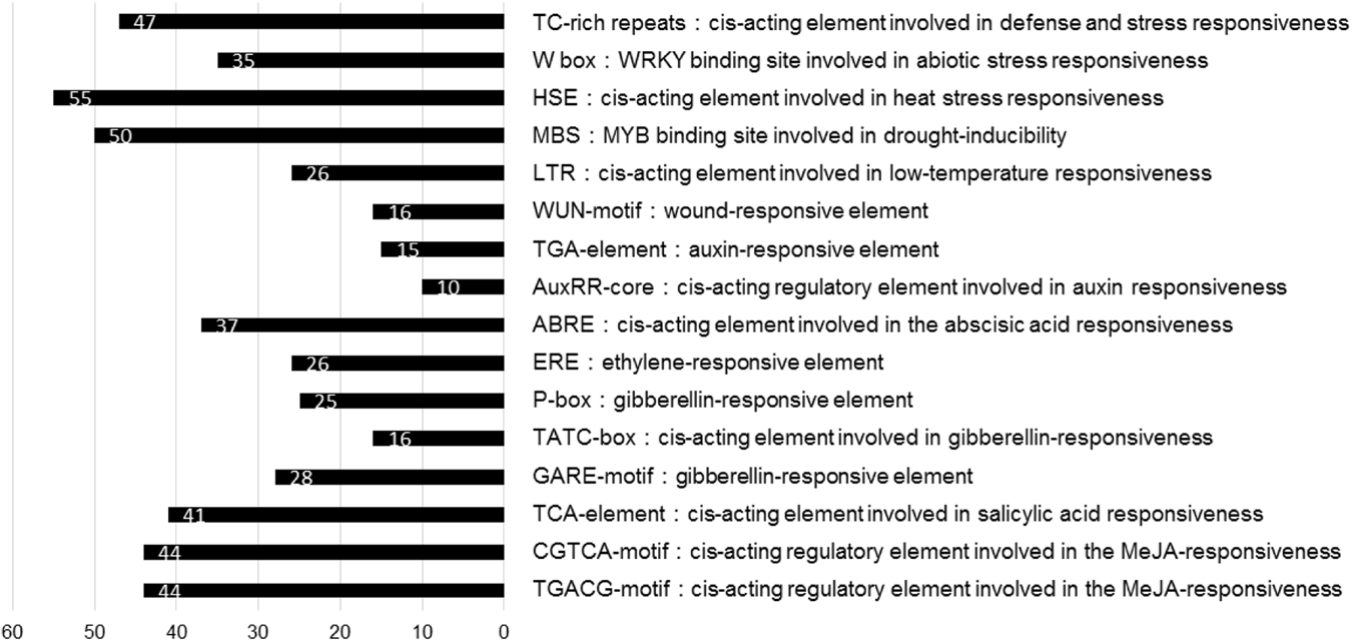
Abiotic stresses and phytohormones response cis-acting elements in *GhSPLs* promoters.

The upstream sequences of 59 *GhSPL* genes (2500 bp upstream of the initiation codon) were used for cis-acting element prediction by PlantCARE^43^. A total of 42 types of putative *cis*-elements involved in light were present in the promoters of *GhSPL*s, including 23 light partial responsive elements (I-box, GAG-motif, GATA-motif, TCT-motif, GA-motif, CATT-motif, TCCC-motif, chs-CMA1a, chs-CMA2a, Gap-box, Box II, LAMP-element, L-box, etc), 6 light responsive elements (Box I, GT1-motif, Sp1, 3-AF1 binding site, MNF1 and AAAC-motif), and other light responsive elements, such as Box 4, G-box, ACE, ATCT-motif, MRE, AE-box, as-2-box, AT1-motif, and ATC-motif (Table S4). Other major cis-elements also include elements responsible to stress response [such as defense and stress (TC-rich repeats), WRKY binding site (W box), heat (HSE), drought (MBS), low-temperature (LTR) and wound (WUN-motif)], and phytohormone response [such as auxin (TGA-element and AuxRR-core), abscisic acid (ABRE), ethylene (ERE), gibberellin (P-box, TATC-box and GARE-motif), salicylic acid (TCA-element) and MeJA (CGTCA-motif and TGACG-motif)] (Fig. 7, Table S4). This suggests the important roles of *GhSPL* genes in biological processes as well as response to abiotic stresses and phytohormones in cotton.

### Expression profiles of SPL genes in *G. hirsutum*

**Figure 8:**
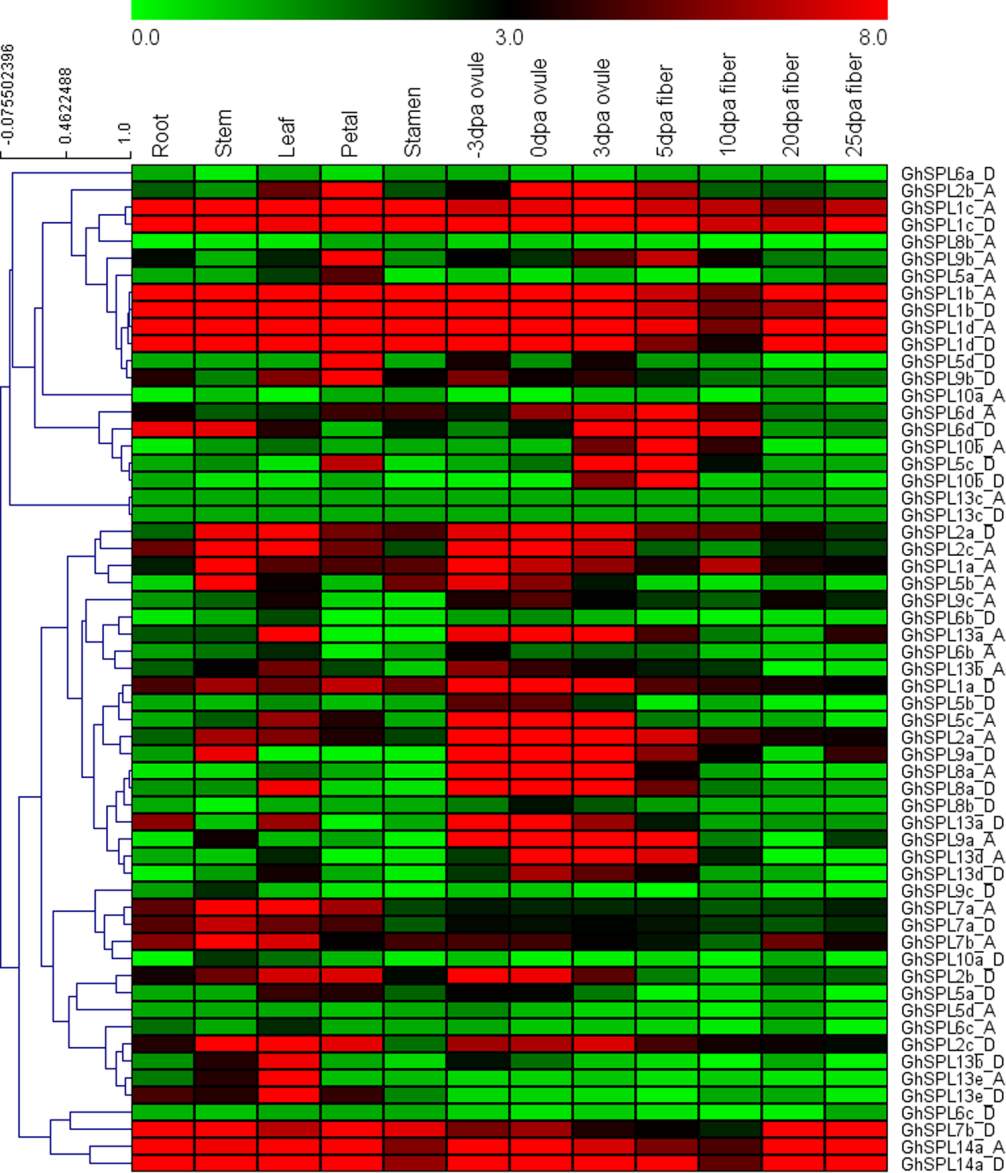
Expression patterns of *GhSPL* genes in different tissues and organs in *G. hirsutum* acc. TM-1. Twelve tissues and organs were root, stem, leaf, petal, stamen, −3, 0 or 3 DPA ovules, 5, 10, 20 and 25 DPA fibers. The color represents *GhSPLs* expression levels: Log2 (FPKM). The phylogenetic relationship was showed on the left.

In order to understand the putative functions of *GhSPL* genes, we analyzed the expression profiles of all the identified 59 SPLs by using the currently available RNA-seq data of *G. hirsutum* acc. TM-1^29^, including 12 different tissues and organs: root, stem, leaf, petal, stamen, −3, 0 or 3 DPA ovules, and 5, 10, 20 or 25 DPA fibers. A heat map expression of *GhSPLs* was showed by Mev4.9.0 in Fig. 8. Nine *SPL* genes *GhSPL1b_A*/*D*, *GhSPL1c_A*/*D*, *GhSPL1d_A*/*D*, *GhSPL14a_A*/*D* and *GhSPL7b_D* were highly expressed in all tissues. Six *GhSPL2* were highly expressed in stem, leaf, petal, −3, 0 and 3 DPA ovules. *GhSPL7a_A*/*D* and *GhSPL7b_A* were highly expressed in stem and leaf. In the *GhSPL5* orthologs, *GhSPL5b_A* were highly expressed in stem and −3 DPA ovules; *GhSPL5c_A* were highly expressed in −3, 0 and 3 DPA ovules; *GhSPL5c_D* were highly expressed in 3 DPA ovules and 5 DPA fibers; *GhSPL5d_D* were highly expressed in petal; *GhSPL5a_A*/*D*, *GhSPL5b_D* and *GhSPL5d_A were* low expressed in all tissues. In the *GhSPL6* orthologs, *GhSPL6d_A*/*D* were highly expressed in 3, 5 and 10 DPA fibers, *GhSPL6a_A*/*D*, *GhSPL6b_A*/*D* and *GhSPL6c_A*/*D were* low expressed in all tissues. In the *GhSPL8* and *GhSPL9* orthologs, *GhSPL8a_A*/*D* and *GhSPL9a_A*/*D* were highly expressed from −3 to 3 DPA ovules; *GhSPL9b_A*/*D* were highly expressed in petal; *GhSPL8b_A*/*D* and *GhSPL9c_A*/*D* were low expressed in all tissues. In the *GhSPL10* orthologs, *GhSPL10b_A*/*D* were highly expressed in 5 DPA fibers; and *GhSPL10a_A*/*D were* low expressed in all tissues. In the *GhSPL13* orthologs, *GhSPL13a_A*/*D* were highly expressed in leaf, −3, 0 and 3 DPA ovules; *GhSPL13b_A*/*D and GhSPL13e_A*/*D were* highly expressed in leaf; *GhSPL13d_A*/*D were* highly expressed in 0 and 3 DPA ovules. However, *GhSPL13c_A*/*D* were no detected in all tested tissues.

## Discussion

In the past couple of years, SPL transcription factors have been attracting attention from the scientific community. Genome-wide identification of SPL gene family has been reported in several plant species. The number of *SPLs* varies from species to species. For instance, there are 15 *SPLs* in potato, pepper, peanut, citrus, *Prunus mume*, danshen, castor bean and tomato^10,13,14,16–19,23^, 17–20 in grape, rice and *Arabidopsis*^22,24^, 27–30 in Chinese cabbage, *Populus* and apple^15,20,21^, 41 in soybean^11^, and 58 in oilseed rape^12^. However, no genome-wide identification of SPL gene family has been reported in cotton although there are four cotton species sequenced. In this study, we reported for the first time the genome-wide identification of SPL genes and systematically investigated the functional structure of SPL transcription factor family. Based on our results, we identified 29, 30 59 and 59 SPL genes in *G. arboretum*, *G. raimondii*, *G. hirsutum* and *G. barbadense*, respectively (Table S1). The number of *SPLs* in A or D genome diploid cotton were similar to *Populus*, and an amount of *SPLs* of allotetraploid cotton species were very close to oilseed rape *B. napus* (AACC, 2n = 38). To compare the number of *SPL* genes and corresponding relationships in four cotton species, we found that there were a typical polyploidization phenomenon. All 29 SPLs in diploid A-genome (*G. arboretum*) and 30 SPLs in D-genome (*G. raimondii*) could be found their homologous genes in allotetraploid genomes (*G. hirsutum* AADD, 20 = 52). Only *SPL6a* was found two homologous genes in four cotton species, *GrSPL6a and GhSPL6a_D* in the D-genome (*G. raimondii*) and D-subgenome of *G. hirsutum*, respectively, and no unique genes were found in the A-genome *G. arboreum*, A-subgenome of *G. hirsutum*, and A- and D- subgenome of *G. barbadense*. Additionally, phylogenetic analysis of SPL proteins in various species showed that green alga were grouped together, and other SPLs were classified into 6–7 groups^21,22^. In this study, phylogenetic tree of 242 SPL proteins from four cotton species (*G. raimondii*, *G. arboretum*, *G. hirsutum* and *G. barbadense*), *A. thaliana*, *O. sativa* and *P. trichocarpa*, showed that all SPL genes were clustered into eight groups (I–VIII) (Fig. 2). Among 177 cotton SPLs from 10 *Arabidopsis* SPLs orthologs, each kinds of orthologs were clustered together.

Segmental duplications play an important role in the gene expansion of SPL transcription factor gene family. Many segmental duplication gene pairs were found in SPL gene family in plants^20–22^. In this study, we identified one pair of tandem duplication (*GrSPL10b*/*GrSPL8b*) and 16 pairs of segmental duplications involved 20 SPL genes in *G. raimondii*. There were 4, 7 and 25 pairs of duplications in *A. thaliana*, *O. sativa* and *P. trichocarpa*, respectively. Additionally, the duplication pattern of SPLs between *G. raimondii* and other three species were analysis; 5, 19 and 22 pairs of duplication events were identified between *G. raimondii* and *O. sativa*, *G. raimondii* and *A. thaliana*, *G. raimondii* and *P. trichocarpa*, respectively. There were 10 ortholog pairs in syntenic regions between *G. raimondii* and *A. thaliana* (Fig. 3, Table S2). Inter-genomic duplication events between *Arabidopsis* and other two species were identified in previous study, including eleven SPLs ortholog pairs between apple and *Arabidopsis*^21^, and nine SPLs ortholog pairs between grape and *Arabidopsis*^22^.

By comparing the number of introns of *SPLs* gene in cotton and *Arabidopsis*, we found that different *SPL* orthologs contained different gene structures, including 9 introns in *SPL1*, *SPL7* and *SPL14*; 2 introns in *SPL6*, *SPL8*, *SPL9*, *SPL10* and *SPL13*; 3 introns in *SPL2* had; 1 introns in *SPL5*. As well as different cotton SPL orthologs shared similar motifs. GhSPL5 had motifs 1 and 2; GhSPL8 had motifs 1, 2 and 14; GhSPL13 had motifs 1, 2 and 15; GhSPL9 and GhSPL10 had motifs 1, 2 14, and 15; GhSPL1, GhSPL2, GhSPL7 and GhSPL14 had more motifs. Among 20 motifs, motif 1 was two Zn-finger like structure, existed in all 59 GhSPL proteins. Motif 2 was nuclear localization signal (NLS), and 56 GhSPLs contained this motif except GhSPL5c_D, GhSPL6a_D and GhSPL9b_D. As showed in Fig. 6B, SBP conserved domain, two zinc finger-like structures and NLS segments, were shared by all GrSPLs. In addition, we speculate that different *SPL* orthologs probably play similar function between cotton and *Arabidopsis*, due to the presence of similar exon/intron structure and conserved motifs.

Several cis-acting elements were found in the promoter regions of *GhSPL*s (Fig. 7, Table S4), which suggests that SPL transcription factors may be regulated by light, stresses and/or phytohormones. All the identified SPL gene show a development- and tissue-dependent expression patterns (Fig. 8). *GhSPL1 and GhSPL14* orthologs were highly expressed in all tested developmental stage and tissues. Some *SPL* genes were highly expressed in certain tissues, and others were low expressed in all tested tissues in the same orthologs. *GhSPL2*, *GhSPL5b_A*/*D*, *GhSPL5c_A*/*D*, *GhSPL6d_A*/*D*, *GhSPL8a_A*/*D*, *GhSPL9a_A*/*D*, *GhSPL10b_A*/*D*, *GhSPL13a_A*/*D*, *GhSPL13d_A*/*D* were highly expressed in −3, 0 and 3 DPA ovules or 5, 10 DPA fibers. This result suggests that cotton SPL gene family may play an important role during fiber initiation, and cotton paralog genes possibly existed subfunctionalization, lost functions, even gained new functions. To date, there are only two expression and function study of SPL genes in cotton^8,26^. Liu *et al*.^8^ reported the expression level of *GhSPLs* and two MADS-box genes (orthologs of *AtAGL6* and *SITDR8*) were repressed in the miR157 over-expression cotton lines. Hypothesized that the miR157/SPL may regulate floral organ size and ovule production in cotton. Zhang *et al*.^26^ reported that *GhSPL3* and *GhSPL18* might be involved in the development of leaves and second shoots, and promoting flowering by overexpression target genes in *Arabidopsis* plants.

**Figure 9:**
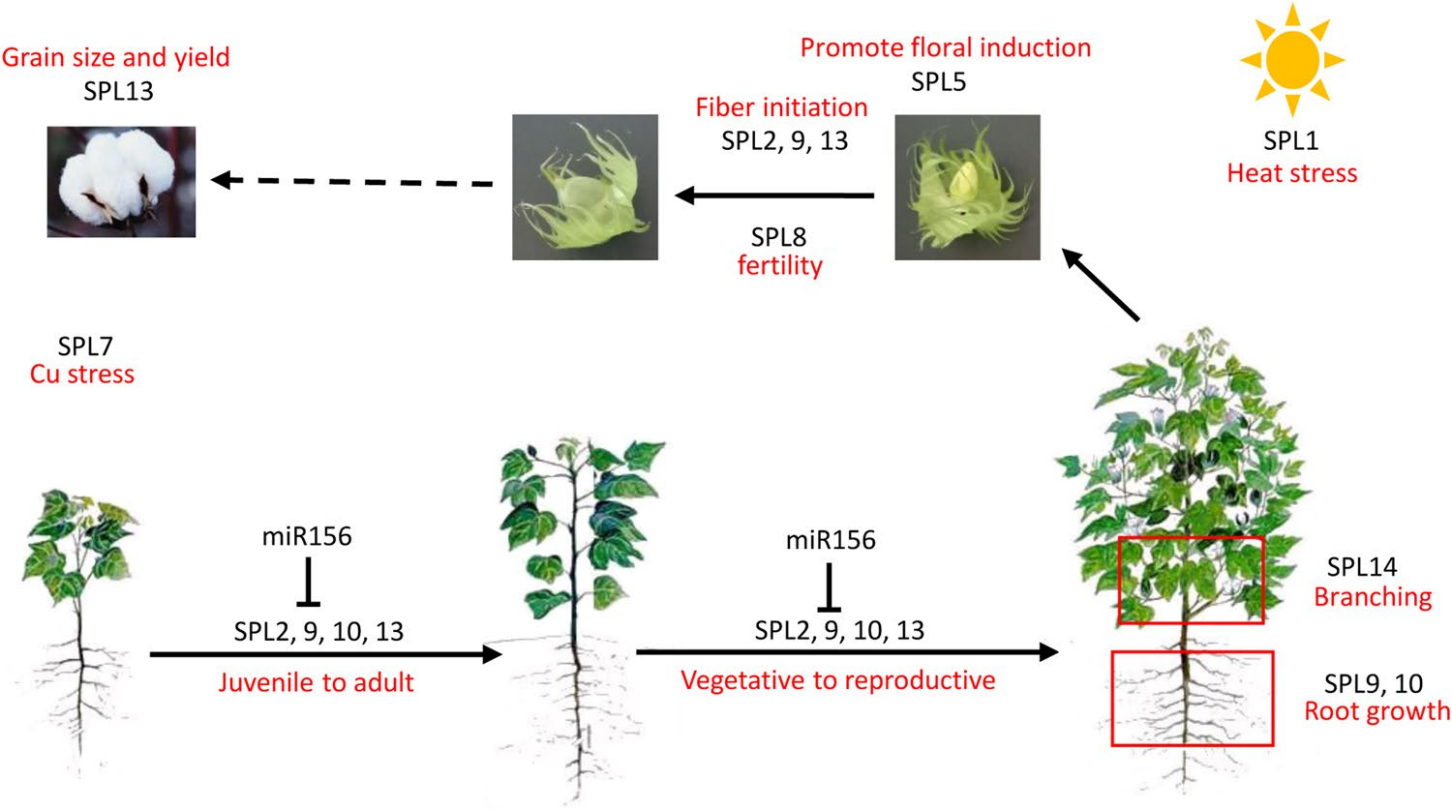
The potential function of SPL orthologs in cotton.

MicroRNAs (miRNAs) may also involve in SPL-regulated gene networks. Among 17 *SPLs* in *Arabidopsis*, 10 were putative targets of miR156/157^4,44^. 11 of 19 *SPLs* in rice^45^, 18 of 28 *SPLs* in *Populus*^20^, 17 of 41 *SPLs* in soybean^11^ were reported to be potential targeted by certain miR156. In this study, we also found that 31 of 59 identified SPLs were potentially targeted by miR156 in upland cotton, which are from 6 different orthologs (*GhSPL2*, *GhSPL6*, *GhSPL9*, *GhSPL10* and *GhSPL13*). Interestingly, motif 7 was existed in those SPLs, and it is a potential target site for the miR156/miR157. In *Arabidopsis* and rice, motif contains miR156 recognition element was also reported in all miR156-targeted SPLs^4,25,45^. Thus, *SPL* gene function analysis mainly through significantly represses the SPL transcriptions by over-expression of miR156/miR157. *Arabidopsis* as an important model plant species, the majority of *AtSPL* genes have been well functionally characterized. SPL2, SPL9, SPL10, SPL11, SPL13 and SPL15 contribute shoot development and the phase transition from vegetative growth to reproductive growth^5^. SPL3, SPL4 and SPL5 primarily promote floral induction and/or floral meristem identity, by SOC1-SPL module control flowering time^46^, and act synergistically with FT-FD module to induce flowering under LDs^47^. SPL3, SPL9 and SPL10 are involved in lateral root growth^48^. SPL1 and SPL12 confer plant thermotolerance at the reproductive stage^49^. SPL7 regulates the Cu deficiency response^50,51^. SPL8 acts in concert to secure male fertility and regulates gynoecium differential patterning^52–54^. Based on the high conservation of SPL gene family between cotton and *Arabidopsis*, we speculate that SPL gene family in cotton may involve in the timing of vegetative and reproductive phase change, root growth, leaf development, fertility, fiber initiation development, response to stresses and yield (Fig. 9). However, the detailed function of each SPL transcription factor in cotton remains to be investigated. This is a genome-wide analysis of SPL gene family in cotton, which will provide the overall and useful information for well functional analysis in the future.

## Materials and Methods

### Sequence sources

The sequences of four sequenced cotton species, *G. raimondii*, *G. arboreum*, *G. hirsutum* acc. TM-1, and *G. barbadense* acc. 3–79, were downloaded from http://www.phytozome.net/, http://cgp.genomics.org.cn, http://mascotton.njau.edu.cn/, and http://cotton.cropdb.org/cotton/download/data.php, respectively. The SPL protein sequence data were obtained for *A. thaliana*, *O. sativa* and *P. trichocarpa* from the Plant Transcription Factor Databases^55^ (Plant TFDB v4.0, planttfdb.cbi.pku.edu.cn/), the General Feature Format (GFF) file *Arabidopsis* Information Resource (TAIR release 10, http://www.arabidopsis.org), the Rice Genome Annotation Project Database (RGAP release 7, http://rice.plantbiology.msu.edu/index.shtml), and (ftp://plantgenie.org/Data/PopGenIE/Populus_trichocarpa/v2.2/), respectively. The gene name and ID were listed in the supplementary materials (Table S5), including 17, 18, and 30 known *SPL* genes in *A. thaliana*, *O. sativa* and *P. trichocarpa*, respectively.

### Identification of SPL transcription factor family in cotton and their chromosomal mapping

SBP domain (PF03110) for SQUAMOSA-PROMOTER BINDING PROTEIN was downloaded from Pfam^56^ (http://pfam.xfam.org/), and was employed to identify all possible *SPL* genes in four cotton species using HMMER (v3.1b2)^35^ (http://hmmer.org) with the e-value < 1e-10. Each candidate SPL gene was further confirmed using SMART^36^ (http://smart.embl-heidelberg.de/) and CDD^37^ (http://www.ncbi.nlm.nih.gov/Structure/cdd/wrpsb.cgi). The theoretical pI (isoelectric point) and molecular weight of the *GhSPLs* were investigated within Expasy^57^ (http://web.expasy.org/protparam/).

The physical location of the *SPL*s in *G. hirsutum* and *G. raimondii* were fetched from the corresponding GFF files. MapInspect (http://mapinspect.software.informer.com/) was used to visualize the distribution of the *SPL* genes in *Gossypium* genome.

### Phylogenetic and gene duplication

A phylogenetic tree was constructed using ClustalW alignment and the Neighbor-Joining (NJ) method in MEGA 7.0 software^39^ (https://mega.nz/), with 1000 replicates boot- strap test. The genome-wide intra- and inter-genomic duplication files of *G. raimondii*, *A. thaliana*, *O. sativa* and *P. trichocarpa* were downloaded from the PGDD^40^ (http://chibba.agtec.uga.edu/duplication), and the visualization was carried out with the CIRCOS tool^58^ (http://circos.ca/). The ratios of Ka/Ks were used to assess the selection pressure for duplication genes.

### Gene structure and conserved motif

The exon/intron structures of *GhSPLs* were drawn using GSDS 2.0^41^ (http://gsds.cbi.pku.edu.cn/), through inputting genes GFF files. MEME (suite 4.11.4)^42^ (http://meme-suite.org/) was employed to identify conserved motifs of GhSPLs with the following parameters: the maximum number of motifs 20, and optimum width from 6 to 250. In addition, SBP domain was presented alone using Multiple alignment program MAFFT version 7 (http://mafft.cbrc.jp/alignment/server/).

### Promoter regions cis-acting elements analysis

The promoter sequences (2500 bp upstream of the initiation codon “ATG”) of 59 *GhSPL* genes were extracted from genome sequences of *G. hirsutum*. The PlantCARE^43^ (http://bioinformatics.psb.ugent.be/webtools/plantcare/html/search_CARE.html) were used to find putative cis-acting elements.

### Expression pattern analysis

To analyze the expression patterns of *GhSPL* genes, we used RNA-seq data of *G. hirsutum* acc. TM-1^29^, including root, stem, leaf, petal, stamen, −3, 0 or 3 DPA ovules, 5, 10, 20 and 25 DPA fibers. The expression levels of *GhSPL* genes were calculated using Log2 (FPKM), fragments per kilobase of exon per million fragments mapped. Expression patterns were display in Mev4.9.0 (https://sourceforge.net/projects/mev-tm4/), and clustered by hierarchical clustering model.

## Electronic supplementary material

Supplementary materials
