# Supplementary material for: Genome-wide identification and characterization of SPL transcription factor family and their evolution and expression profiling analysis in cotton
Source: Sci Rep. 2018 Jan 15;8:762. doi: 10.1038/s41598-017-18673-4 (PMC5768680; doi:10.1038/s41598-017-18673-4)

Genome-wide identification and characterization of SPL transcription factor family and their evolution and expression profiling analysis in cotton

Caiping Cai [1,2,*], Wangzhen Guo [1] & Baohong Zhang [2,*]

Running title: SPLs in cotton

1 State Key Laboratory of Crop Genetics & Germplasm Enhancement, Hybrid Cotton R & D Engineering Research Center, Ministry of Education, Nanjing Agricultural University, Nanjing 210095, China. 2 Department of Biology, East Carolina University, Greenville, NC 27858, USA. Correspondence and requests for materials should be addressed to C.C. (email: cpcai@njau.edu.cn) or B.Z. (email: zhangb@ecu.edu)

Supplementary Table S1. Characterization of SLP family genes identified in *Gossypium*.

**Gene name***	**Chr.**	**Gene ID in *G. hirsutum***	**Domain**	**Length (AA)** [b]	**pI**	**MW (Da)**	***G. arboreum* / *G.raimondii***	***G. barbadense***
GhSPL1a_A	A02	*Gh_A02G0579*	SBP, ANK	1041	7.8	115940.63	*GaSPL1a* (Cotton_A_08761)	*GbSPL1a_A* (Gbscaffold2519.11.0)
GhSPL1a_D	D02	*Gh_D02G0637*	SBP, ANK	1040	8.06	115688.35	*GrSPL1a* (Gorai.005G071400)	*GbSPL1a_D* (Gbscaffold14670.7.0)
GhSPL1b_A	A08	*Gh_A08G0326*	SBP, ANK	982	5.94	109019.76	*GaSPL1b* (Cotton_A_12618)	*GbSPL1b_A* (Gbscaffold2358.10.0)
GhSPL1b_D	D08	*Gh_D08G0423*	SBP, ANK	982	5.86	108875.42	*GrSPL1b* (Gorai.004G047200)	*GbSPL1b_D* (Gbscaffold5399.26.0)
GhSPL1c_A	A11	*Gh_A11G0943*	SBP, ANK	985	5.86	109050.73	*GaSPL1c* (Cotton_A_15677)	*GbSPL1c_A* (Gbscaffold2627.11.0)
GhSPL1c_D	D11	*Gh_D11G1088*	SBP, ANK	985	6.02	109027.78	*GrSPL1c* (Gorai.007G116100)	*GbSPL1c_D* (Gbscaffold209.5.0)
GhSPL1d_A	A12	*Gh_A12G1096*	SBP, ANK	987	6.14	109727.83	*GaSPL1d* (Cotton_A_22632)	*GbSPL1d1_A* (Gbscaffold21079.4.0), *GbSPL1d2_A* (Gbscaffold21079.3.0)
GhSPL1d_D	D12	*Gh_D12G1220*	SBP, ANK	987	6.02	109591.69	*GrSPL1d* (Gorai.008G135700)	*GbSPL1d_D* (Gbscaffold998.36.0)
GhSPL2a_A	A01	*Gh_A01G1281*	SBP	477	7.66	52795.41	*GaSPL2a* (Cotton_A_38223)	*GbSPL2a_A* (Gbscaffold4848.1.0)
GhSPL2a_D	D01	*Gh_D01G1503*	SBP, Alpha_adaptinC2	580	8.32	63964.67	*GrSPL2a* (Gorai.002G182700)	*GbSPL2a_D* (Gbscaffold2427.17.0)
GhSPL2b_A	A03	*Gh_A03G1092*	SBP	473	8.55	52252.33	*GaSPL2b* (Cotton_A_33286)	*GbSPL2b_A* (Gbscaffold4888.4.0)
GhSPL2b_D	D02	*Gh_D02G1515*	SBP	474	7.97	52187.16	*GrSPL2b* (Gorai.005G166500)	*GbSPL2b_D* (Gbscaffold10092.5.0)
GhSPL2c_A	A04	*Gh_A04G1486*	SBP	482	7.62	53414.7	*GaSPL2c* (Cotton_A_14124)	*GbSPL2c_A* (Gbscaffold27493.6.0)
GhSPL2c_D	D04	*Gh_D04G1827*	SBP	476	7.65	52597.93	*GrSPL2c* (Gorai.012G176500)	*GbSPL2c_D* (Gbscaffold15478.47.0)
GhSPL5a_A	A01	*Gh_A01G0447*	SBP	182	8.63	20195.49	*GaSPL5a* (Cotton_A_06263)	*GbSPL5a_A* (Gbscaffold4503.10.0)
GhSPL5a_D	D01	*Gh_D01G0457*	SBP	182	8.85	20260.55	*GrSPL5a* (Gorai.002G068300)	*GbSPL5a_D* (Gbscaffold6672.5.0)
GhSPL5b_A	A02	*Gh_A02G1694*	SBP	174	8.97	20291.65	*GaSPL5b* (Cotton_A_01498)	/
GhSPL5b_D	D03	*Gh_D03G0025*	SBP	177	8.14	20594.87	*GrSPL5b* (Gorai.003G003200)	*GbSPL5b_D* (Gbscaffold1758.17.0)
GhSPL5c_A	A10	*Gh_A10G2217*	SBP	141	8.17	16111.77	*GaSPL5c* (Cotton_A_03916)	*GbSPL5c1_A* (Gbscaffold10272.19.0)
GhSPL5c_D	D10	*Gh_D10G0251*	SBP	154	6.95	16964.98	*GrSPL5c* (Gorai.011G029400)	*GbSPL5c2_A* (Gbscaffold9936.11.0)
GhSPL5d_A	A13	*Gh_A13G1251*	SBP	182	9.08	20417.79	*GaSPL5d* (Cotton_A_16783)	/
GhSPL5d_D	D13	*Gh_D13G1551*	SBP	182	9.08	20417.79	*GrSPL5d* (Gorai.013G169800)	*GbSPL5d_D* (Gbscaffold12691.1.0)
GhSPL6a_D	D02	*Gh_D02G1306*	SBP	205	8.1	22909.88	*GrSPL6a* (Gorai.005G146800)	/
GhSPL6b_A	A03	*Gh_A03G0632*	SBP	525	6.39	57148.28	*GaSPL6b* (Cotton_A_34368)	*GbSPL6b_A* (Gbscaffold2648.18.0)
GhSPL6b_D	D03	*Gh_D03G0920*	SBP	525	6.53	57077.26	*GrSPL6b* (Gorai.003G102800)	*GbSPL6b_D* (Gbscaffold13521.2.0)
GhSPL6c_A	A04	*Gh_A04G1266*	SBP	379	7.2	42592.6	*GaSPL6c* (Cotton_A_11747)	/

GhSPL6c_D	D04	*Gh_D04G1893*	SBP	379	7.2	42632.72	*GrSPL6c* (Gorai.012G185700)	*GbSPL6c_D* (Gbscaffold12316.2.0)
GhSPL6d_A	A13	*Gh_A13G0749*	SBP	503	7.93	55705.35	*GaSPL6d* (Cotton_A_38193)	*GbSPL6d_A* (Gbscaffold2116.7.0)
GhSPL6d_D	D13	*Gh_D13G0874*	SBP	399	9.01	43752.34	*GrSPL6d* (Gorai.013G095700)	/
GhSPL7a_A	A02	*Gh_A02G0446*	SBP	810	6.3	90582.96	*GaSPL7a1* (Cotton_A_21628), *GaSPL7a2* (Cotton_A_30087)	*GbSPL7a1_A* (Gbscaffold6713.1.0), *GbSPL7a2_A* (Gbscaffold6713.2.0)
GhSPL7a_D	D02	*Gh_D02G0498*	SBP	804	6.05	89816.03	*GrSPL7a* (Gorai.005G056500)	*GbSPL7a_D* (Gbscaffold5562.17.0)
GhSPL7b_A	A10	*Gh_A10G0934*	SBP, NPY1	793	6.24	89789.37	*GaSPL7b* (Cotton_A_32103)	*GbSPL7b_A* (Gbscaffold8838.1.0)
GhSPL7b_D	D10	*Gh_D10G1634*	SBP, NPY1	793	6.13	89616.21	*GrSPL7b* (Gorai.011G183800)	*GbSPL7b_D* (Gbscaffold3108.1.0)
GhSPL8a_A	A07	*Gh_A07G1375*	SBP	369	8.92	41498.82	*GaSPL8a* (Cotton_A_31831)	*GbSPL8a_A* (Gbscaffold10961.2.0)
GhSPL8a_D	D07	*Gh_D07G1479*	SBP	290	8.91	32631.88	*GrSPL8a* (Gorai.001G176000)	*GbSPL8a_D* (Gbscaffold42636.1.0)
GhSPL8b_A	A12	*Gh_A12G0867*	SBP	289	8.73	32305.46	*GaSPL8b* (Cotton_A_31207)	*GbSPL8b_A* (Gbscaffold4809.4.0)
GhSPL8b_D	D12	*Gh_D12G0948*	SBP	289	8.73	32263.38	*GrSPL8b* (Gorai.008G107400)	*GbSPL8b_D* (Gbscaffold140.1.0)
GhSPL9a_A	A01	*Gh_A01G2095*	SBP	374	9.13	40445.05	*GaSPL9a* (Cotton_A_39554)	*GbSPL9a_A* (Gbscaffold5683.4.0)
GhSPL9a_D	D01	*Gh_D01G1229*	SBP	280	9.3	30060.43	*GrSPL9a* (Gorai.002G156700)	*GbSPL9a_D* (Gbscaffold635.1.0)
GhSPL9b_A	A04	*Gh_A04G1331*	SBP, Clr2_transil	389	8.85	41290.7		*GbSPL9b1_D* (Gbscaffold13517.3.0), *GbSPL9b2_D* (Gbscaffold3935.1.0)
GhSPL9b_D	D04	*Gh_D04G1985*	SBP	352	7.6	37193.97	*GrSPL9b* (Gorai.006G066700)	*GbSPL9b_A* (Gbscaffold13517.4.0)
GhSPL9c_A	A11	*Gh_A11G2811*	SBP	347	9.03	37848.17	*GaSPL9c* (Cotton_A_06609)	*GbSPL9c1_A* (Gbscaffold15997.13.0), *GbSPL9c2_A* (Gbscaffold7329.32.0)
GhSPL9c_D	D11	*Gh_D11G3165*	SBP	347	9.04	38083.45	*GrSPL9c* (Gorai.007G362000)	*GbSPL9c_D* (Gbscaffold15997.12.0)
GhSPL10a_A	A11	*Gh_A11G0706*	SBP	420	7.32	45912.31	*GaSPL10a* (Cotton_A_07701)	*GbSPL10a_A* (Gbscaffold19628.3.0)
GhSPL10a_D	D11	*Gh_D11G0821*	SBP	420	7.7	45925.23	*GrSPL10a* (Gorai.007G087900)	*GbSPL10a_D* (Gbscaffold3124.18.0)
GhSPL10b_A	A12	*Gh_A12G0866*	SBP	358	8.64	40079.55	*GaSPL10b* (Cotton_A_31206)	*GbSPL10b_A* (Gbscaffold4809.6.0)
GhSPL10b_D	D12	*Gh_D12G0947*	SBP	359	8.3	40136.42	*GrSPL10b* (Gorai.008G107300)	*GbSPL10b_D* (Gbscaffold13585.2.0)
GhSPL13a_A	A01	*Gh_A01G1274*	SBP	281	9.37	30978.7	*GaSPL13a* (Cotton_A_03328)	*GbSPL13a_A* (Gbscaffold1620.5.0)
GhSPL13a_D	D01	*Gh_D01G1495*	SBP	290	9.43	32085.98	*GrSPL13a* (Gorai.002G181700)	*GbSPL13a1_D* (Gbscaffold1620.4.0), *GbSPL13a2_D* (Gbscaffold17331.2.0)
GhSPL13b_A	A03	*Gh_A03G1083*	SBP	309	9.88	33489.39	*GaSPL13b* (Cotton_A_22535)	*GbSPL13b_A* (Gbscaffold874.1.0)
GhSPL13b_D	D02	*Gh_D02G1499*	SBP	309	9.83	33467.36	*GrSPL13b* (Gorai.005G164600)	*GbSPL13b_D* (Gbscaffold9618.6.0)
GhSPL13c_A	A07	*Gh_A07G0442*	SBP	351	9.13	39388.28	*GaSPL13c* (Cotton_A_19943)	*GbSPL13c_A* (Gbscaffold1020.25.0)
GhSPL13c_D	D07	*Gh_D07G0506*	SBP	352	9.41	39662.54	*GrSPL13c* (Gorai.001G057700)	*GbSPL13c_D* (Gbscaffold7269.22.0)
GhSPL13d_A	A11	*Gh_A11G0344*	SBP	354	9.24	39628.91	*GaSPL13d* (Cotton_A_01939)	*GbSPL13d_D* (Gbscaffold21103.38.0)
GhSPL13d_D	D11	*Gh_D11G0401*	SBP	424	8.98	47235.45	*GrSPL13d* (Gorai.007G043500)	*GbSPL13d_A* (Gbscaffold7307.4.0)

GhSPL13e_A	A12	*Gh_A12G1380*	SBP	302	8.42	33131.72	*GaSPL13e* (Cotton_A_20189)	*GbSPL13e_A* (Gbscaffold462.5.0)
GhSPL13e_D	D12	*Gh_D12G1504*	SBP	302	8.42	33271.9	*GrSPL13e* (Gorai.008G166000)	*GbSPL13e_D* (Gbscaffold6335.4.0)
GhSPL14a_A	A01	*Gh_A01G0812*	SBP, ANK	1083	8.75	119712.83	*GaSPL14a* (Cotton_A_12693)	*GbSPL14a_D* (Gbscaffold12297.8.0)
GhSPL14a_D	D01	*Gh_D01G0839*	SBP, ANK	1083	8.8	119882.05	*GrSPL14a* (Gorai.002G112200)	*GbSPL14a_A* (Gbscaffold789.1.0)

*** All 31 miR156-targeted SPLs were indicated in red.**

Supplementary Table S2. Intra- or inter-genome duplications of *SPL* genes in *G. raimondii* and other three species.

Species-Species*	ID 1	Gene 1	ID 2	Gene 2	Ka	Ks
at-gr	AT5G50670	*AtSPL13B*	Gorai.007G043500	*GrSPL13d*	0.6089	1.9012
at-gr	AT5G18830	*AtSPL7*	Gorai.005G056500	*GrSPL7a*	0.4009	2.0009
at-gr	AT5G18830	*AtSPL7*	Gorai.011G183800	*GrSPL7b*	0.4146	1.6339
at-gr	AT5G50670	*AtSPL13B*	Gorai.001G057700	*GrSPL13c*	0.7952	2.425
at-gr	AT3G57920	*AtSPL15*	Gorai.007G362000	*GrSPL9c*	0.6185	1.8601
at-gr	AT3G57920	*AtSPL15*	Gorai.002G156700	*GrSPL9a*	0.5207	1.831
at-gr	AT3G15270	*AtSPL5*	Gorai.002G068300	*GrSPL5a*	0.4152	4.2966
at-gr	AT3G15270	*AtSPL5*	Gorai.013G169800	*GrSPL5d*	0	0
at-gr	AT3G15270	*AtSPL5*	Gorai.011G029400	*GrSPL5c*	0	0
at-gr	AT2G47070	*AtSPL1*	Gorai.008G135700	*GrSPL1d*	0.2342	1.6583
at-gr	AT2G42200	*AtSPL9*	Gorai.007G362000	*GrSPL9c*	0.4515	1.7681
at-gr	AT2G42200	*AtSPL9*	Gorai.002G156700	*GrSPL9a*	0.4341	1.9695
at-gr	AT2G33810	*AtSPL3*	Gorai.011G029400	*GrSPL5c*	0	0
at-gr	AT1G27360	*AtSPL11*	Gorai.005G166500	*GrSPL2b*	0.4779	1.4525
at-gr	AT1G76580	*AtSPL16*	Gorai.002G112200	*GrSPL14a*	0.3233	1.4974
at-gr	AT1G53160	*AtSPL4*	Gorai.002G068300	*GrSPL5a*	0.4332	1.9271
at-gr	AT1G20980	*AtSPL14*	Gorai.002G112200	*GrSPL14a*	0.2888	1.6682
at-gr	AT1G27360	*AtSPL11*	Gorai.002G182700	*GrSPL2a*	0.5005	1.5473
at-gr	AT1G69170	*AtSPL6*	Gorai.012G185700	*GrSPL6c*	0.7243	1.9721
gr-gr	Gorai.001G057700	*GrSPL13c*	Gorai.005G164600	*GrSPL13b*	0.676	-1
gr-gr	Gorai.001G057700	*GrSPL13c*	Gorai.007G043500	*GrSPL13d*	0.3642	0.81
gr-gr	Gorai.002G068300	*GrSPL5a*	Gorai.011G029400	*GrSPL5c*	0.2833	1.7426
gr-gr	Gorai.002G182700	*GrSPL2a*	Gorai.012G176500	*GrSPL2c*	0.1846	0.4948
gr-gr	Gorai.002G181700	*GrSPL13a*	Gorai.005G164600	*GrSPL13b*	0.2082	0.4783
gr-gr	Gorai.002G182700	*GrSPL2a*	Gorai.005G166500	*GrSPL2b*	0.1153	0.3987
gr-gr	Gorai.002G181700	*GrSPL13a*	Gorai.008G166000	*GrSPL13e*	0.2319	0.5229
gr-gr	Gorai.003G102800	*GrSPL6b*	Gorai.013G095700	*GrSPL6d*	0.4581	1.3523
gr-gr	Gorai.005G056500	*GrSPL7a*	Gorai.011G183800	*GrSPL7b*	0.1525	0.4235
gr-gr	Gorai.005G166500	*GrSPL2b*	Gorai.012G176500	*GrSPL2c*	0.1806	0.5397
gr-gr	Gorai.005G146800	*GrSPL6a*	Gorai.013G095700	*GrSPL6d*	0.1952	0.5956
gr-gr	Gorai.005G164600	*GrSPL13b*	Gorai.008G166000	*GrSPL13e*	0.1936	0.4793
gr-gr	Gorai.005G071400	*GrSPL1a*	Gorai.008G135700	*GrSPL1d*	0.2896	1.4647
gr-gr	Gorai.007G087900	*GrSPL10a*	Gorai.008G107300	*GrSPL10b*	0.1961	0.7089
gr-gr	Gorai.007G116100	*GrSPL1c*	Gorai.008G135700	*GrSPL1d*	0.0893	0.3665
gr-gr	Gorai.008G166000	*GrSPL13e*	Gorai.012G176500	*GrSPL2c*	0.9104	4.0298
gr-os	Gorai.008G107300	*GrSPL10b*	LOC_Os04g46580	*OsSPL7*	0.694	0
gr-os	Gorai.008G135700	*GrSPL1d*	LOC_Os01g18850	*OsSPL1*	0.5224	2.2553
gr-os	Gorai.007G087900	*GrSPL10a*	LOC_Os04g46580	*OsSPL7*	0.5662	0
gr-os	Gorai.007G116100	*GrSPL1c*	LOC_Os01g18850	*OsSPL1*	0.5235	2.4412

gr-os	Gorai.001G176000	*GrSPL8a*	LOC_Os04g46580	*OsSPL7*	0.8597	0
gr-pt	Gorai.008G107300	*GrSPL10b*	Potri.014G057700	*PtSPL26*	0.7511	2.477
gr-pt	Gorai.008G107300	*GrSPL10b*	Potri.002G142200	*PtSPL21*	0	0
gr-pt	Gorai.007G087900	*GrSPL10a*	Potri.014G057700	*PtSPL26*	0.9519	2.9058
gr-pt	Gorai.007G087900	*GrSPL10a*	Potri.002G142400	*PtSPL8*	0.2884	1.752
gr-pt	Gorai.005G056500	*GrSPL7a*	Potri.010G026200	*PtSPL3*	0.3117	1.1899
gr-pt	Gorai.005G146800	*GrSPL6a*	Potri.010G154300	*PtSPL13*	0.3568	1.0721
gr-pt	Gorai.005G056500	*GrSPL7a*	Potri.008G197000	*PtSPL4*	0.3212	1.2138
gr-pt	Gorai.005G146800	*GrSPL6a*	Potri.008G097900	*PtSPL12*	0.3674	1.2684
gr-pt	Gorai.003G102800	*GrSPL6b*	Potri.015G060400	*PtSPL28*	0.3057	1.0152
gr-pt	Gorai.003G102800	*GrSPL6b*	Potri.010G154300	*PtSPL13*	0.5094	1.268
gr-pt	Gorai.003G102800	*GrSPL6b*	Potri.008G097900	*PtSPL12*	0.4558	0.9731
gr-pt	Gorai.002G068300	*GrSPL5a*	Potri.011G055900	*PtSPL16*	0.3208	1.3904
gr-pt	Gorai.002G068300	*GrSPL5a*	Potri.011G116800	*PtSPL25*	0.2293	1.5601
gr-pt	Gorai.002G068300	*GrSPL5a*	Potri.001G398200	*PtSPL20*	0.2504	1.5407
gr-pt	Gorai.013G169800	*GrSPL5d*	Potri.011G116800	*PtSPL25*	0.1973	1.535
gr-pt	Gorai.013G169800	*GrSPL5d*	Potri.011G116800	*PtSPL25*	0.1973	1.535
gr-pt	Gorai.013G095700	*GrSPL6d*	Potri.010G154300	*PtSPL13*	0.3416	1.0092
gr-pt	Gorai.013G095700	*GrSPL6d*	Potri.008G097900	*PtSPL12*	0.2769	0.8686
gr-pt	Gorai.013G169800	*GrSPL5d*	Potri.001G398200	*PtSPL20*	0.2389	1.4875
gr-pt	Gorai.011G183800	*GrSPL7b*	Potri.010G026200	*PtSPL3*	0.3213	1.2944
gr-pt	Gorai.011G183800	*GrSPL7b*	Potri.008G197000	*PtSPL4*	0.3325	1.4259
gr-pt	Gorai.001G176000	*GrSPL8a*	Potri.002G142200	*PtSPL21*	0.3601	1.9574
at-at	AT2G33810	*AtSPL3*	AT3G15270	*AtSPL5*	0.4206	0
at-at	AT2G42200	*AtSPL9*	AT3G57920	*AtSPL15*	0.3141	0.8888
at-at	AT1G53160	*AtSPL4*	AT3G15270	*AtSPL5*	0.1883	0.5755
at-at	AT1G20980	*AtSPL14*	AT1G76580	*AtSPL16*	0.1475	0.6375
os-os	LOC_Os01g69830	*OsSPL2*	LOC_Os08g41940	*OsSPL16*	0.5647	0.8302
os-os	LOC_Os01g69830	*OsSPL2*	LOC_Os09g32944	*OsSPL18*	0.7888	0.8856
os-os	LOC_Os02g04680	*OsSPL3*	LOC_Os06g49010	*OsSPL12*	0.322	0.938
os-os	LOC_Os02g07780	*OsSPL4*	LOC_Os06g45310	*OsSPL11*	0.4237	0.9115
os-os	LOC_Os02g08070	*OsSPL5*	LOC_Os06g44860	*OsSPL10*	0.4502	0.5965
os-os	LOC_Os08g39890	*OsSPL14*	LOC_Os09g31438	*OsSPL17*	0.2122	0.6828
os-os	LOC_Os08g41940	*OsSPL16*	LOC_Os09g32944	*OsSPL18*	0.2516	0.6908
pt-pt	Potri.012G100700	*PtSPL15*	Potri.015G098900	*PtSPL14*	0.1123	0.2898
pt-pt	Potri.011G055900	*PtSPL16*	Potri.011G116800	*PtSPL25*	0.251	1.9323
pt-pt	Potri.010G154000	*PtSPL1*	Potri.014G114300	*PtSPL6*	0.2938	1.083
pt-pt	Potri.008G098600	*PtSPL5*	Potri.014G114300	*PtSPL6*	0.2793	1.2222
pt-pt	Potri.008G097900	*PtSPL12*	Potri.010G154300	*PtSPL13*	0.1031	0.2374
pt-pt	Potri.008G098600	*PtSPL5*	Potri.010G154000	*PtSPL1*	0.0654	0.2211
pt-pt	Potri.008G197000	*PtSPL4*	Potri.010G026200	*PtSPL3*	0.0837	0.2211
pt-pt	Potri.004G046700	*PtSPL23*	Potri.017G012900	*PtSPL30*	0.7134	1.3108
pt-pt	Potri.004G046700	*PtSPL23*	Potri.011G116800	*PtSPL25*	0.2938	2.0124

pt-pt	Potri.004G046700	*PtSPL23*	Potri.011G055900	*PtSPL16*	0.1116	0.2518
pt-pt	Potri.003G169400	*PtSPL22*	Potri.015G098900	*PtSPL14*	0.4217	1.6957
pt-pt	Potri.003G169400	*PtSPL22*	Potri.012G100700	*PtSPL15*	0.5148	1.7763
pt-pt	Potri.002G142400	*PtSPL8*	Potri.014G057800	*PtSPL27*	0.0734	0.2857
pt-pt	Potri.002G142200	*PtSPL21*	Potri.014G057700	*PtSPL26*	0.075	0.3126
pt-pt	Potri.002G188700	*PtSPL7*	Potri.014G114300	*PtSPL6*	0.0517	0.2408
pt-pt	Potri.002G188700	*PtSPL7*	Potri.010G154000	*PtSPL1*	0.288	1.2121
pt-pt	Potri.002G188700	*PtSPL7*	Potri.008G098600	*PtSPL5*	0.2751	1.3711
pt-pt	Potri.002G002400	*PtSPL2*	Potri.005G258700	*PtSPL9*	0.0649	0.2606
pt-pt	Potri.001G058600	*PtSPL18*	Potri.015G098900	*PtSPL14*	0.4384	1.6494
pt-pt	Potri.001G058600	*PtSPL18*	Potri.012G100700	*PtSPL15*	0.5117	1.7448
pt-pt	Potri.001G398200	*PtSPL20*	Potri.011G055900	*PtSPL16*	0	0
pt-pt	Potri.001G398200	*PtSPL20*	Potri.011G116800	*PtSPL25*	0.0764	0.3417
pt-pt	Potri.001G398200	*PtSPL20*	Potri.004G046700	*PtSPL23*	0	0
pt-pt	Potri.001G058600	*PtSPL18*	Potri.003G169400	*PtSPL22*	0.0619	0.175
pt-pt	Potri.001G055900	*PtSPL19*	Potri.003G172600	*PtSPL11*	0.1506	0.2414

***At, Os, Pt and Gr indicated A. thaliana, O. sativa, P. trichocarpa and G. raimondii, respectively.**

Supplementary Table S3. The length and consensus sequence of 20 motifs identified in *GhSPLs*.

Motif	Length	Consensus sequence
1	49	PPRCQVEGCNADLSDAKDYHRRHKVCEMHSKAPKVIVAGIEQRFCQQCS
2	29	RFHVLSEFDEGKRSCRRRLAGHNERRRKP
3	80	AQCRTDRIVFKLFGWNPNDFPLVLRHQIYDWLSHMPTDIESYIRPGCIILTIYICMPEAAWDELCYNLMFYMHRLVDCSD
4	159	WLLHRSQLKSRLGHLDPNPELFPLRRFKWLMEFSMDHEWCAVVKKLLNILLDGTVGLGEHPSMNLALTEMCLLHRAV RKNCRPLVELLLRFIPEKTSDKLGFENEMVADGVHYSFLFRPDVIGPAGLTPLHIAAGKDGCEDVLDALTDDPGKVGIDA WK
5	113	DTFWRTGWICIRVQDQIAFIYNGQVVVDTYLPLGSNHYCKIMSVKPIAVCATERAQFSVKGINLSQPATRLLCAVEGKYL VQEATHELMDDNDDFKEQDELQCINFSCSIPTV
6	158	RMLYGRGFMTIYLNNMIFRTRKGGTSMVKIDMGVQVPRLHYVYPACFEAGKPMEFVACGSNWLQPKFQFLVSFAGRY LPYYYCVASAHVQATDGSPSCDHQLYKIYIPQTEPDLFGPVFIEVENQSGLSNFIPVLIGDKDVCSEMKVIQQGFDASVFQ
7	50	NMSLVYRPAMLSMVAIAAVCVCVALLFKSCPEVLYVFRPFRWELLDYGTS
8	70	NPDAVVNGNSLNDEQTSGYLLLSLLKILSNMHSNRSDQTTDQDVLTHLLRSLANHTCEQGGRNISGLLPE
9	51	DLKAVGKRTVEWDLNDWKWDGDLFIASPINPVSADCMGRQFFPLGSGIPGN
10	113	AVSALFSNGQGPPRPFKQHITGPASEMPQKGVHSHDTRGAEVQGNAAGAVKMNNFDLNDIYIDSDDETDDIERFPAPVN MGTSSLDCPSWVQQDSHQSSPPQTSRNSDSASAQ
11	33	AQDSTGSTPEDYARLRGHYSYIHLVQKKINKRH
12	73	GKQQMSFVWNKVPFLHNARPNEIFTWEGTFYSKSSQMKGYTPTKVGNINGQPQLPGNQLLNPITMRCHDFNKF
13	130	CETSTLQHKAYSEFVLDIAWLLREPKLENFKETMASSQIQRFNCLLNFLIQNESTVILKKILQNLKIVVEMIGFDGTDDPD VRLLKKYMDYARDILSNELQEGEIPVFRSEYIEQEGKWNSQSSFDNDGL
14	29	VASNSDDEPLICLKLGKRTYFEDATDGFN
15	21	VQDSDCALSLLSNQPWEWREI
16	42	IEDHCYNSSFFPFIVAEDDVCSEIRMLESVLETTDTDADIGR
17	57	ELEKKRRVTVVEDDSLNEEAGSLTLKLGGQGGHGYPISQREMKNWEGTSGKKTKLSG
18	41	TNQKQNNESTSSFEIGQLELRPIKRHCKLCDQKLAYGYGTA
19	57	LGLASLSKSSKSASINSSSMGEVKMTKFTLEAFEAIPDDISNKKEVFKIEHTGTSPT
20	57	GPDRVRKRDPRMTCSNFIAGRVPCACPEIDEQMEKLEEEEAGAPGKKRARTGRVGSG

Supplementary Table S4. Cis-acting elements of *GhSPL* genes promoter regions.

Element	Core sequence	Function annotation	No. of genes containing element
CAAT-box	CAAAT/CAAT	common cis-acting element in promoter and enhancer regions	59
TATA-box		core promoter element around -30 of transcription start	59
I-box	CTCTTATGCT	part of a light responsive element	44
GAG-motif	GGAGATG	part of a light responsive element	37
GATA-motif	AAGGATAAGG	part of a light responsive element	36
TCT-motif	TCTTAC	part of a light responsive element	35
GA-motif	ATAGATAA	part of a light responsive element	31
CATT-motif	GCATTC	part of a light responsive element	23
TCCC-motif	TCTCCCT	part of a light responsive element	20
chs-CMA1a	TTACTTAA	part of a light responsive element	13
chs-CMA2a	TCACTTGA	part of a light responsive element	9
Gap-box	AAATGGAGA	part of a light responsive element	9
Box II	TCCACGTGGC	part of a light responsive element	8
LAMP-element	CTTTATCA	part of a light responsive element	8
L-box	CTCACCTACCAA	part of a light responsive element	7
rbcS-CMA7a	GTCGATAAGG	part of a light responsive element	4
chs-Unit 1 m1	ACCTACCACAC	part of a light responsive element	3
CG-motif	CCATGGGG	part of a light responsive element	3
LS7	CAGATTTATTTTTA	part of a light responsive element	2
chs-CMA2b	ATTGCAACTCAA	part of a light responsive element	2
GATT-motif	CTGCAGATTTCT	part of a light responsive element	2
TGGCA-motif	GATGGAAGTGGCA	part of a light responsive element	2
Pc-CMA2a	CAACCAATGAAAA	part of a light responsive element	1
sbp-CMA1c	CTTTATCTCTTCCA	part of a light responsive element	1
TGG-motif	GGTTGCCA	part of a light responsive element	1
Box I	TTTCAAA	light responsive element	52
GT1-motif	GGTTAA/GGTTAAT	light responsive element	47
Sp1	CC(G/A)CCC	light responsive element	37
3-AF1 binding site	AAGAGATATTT	light responsive element	14
MNF1	GTGCCC(A/T)(A/T)	light responsive element	6
AAAC-motif	CAATCAAAACCT	light responsive element	3
Box 4	ATTAAT	part of a conserved DNA module involved in light responsiveness	58
G-box	CACGTA/TACGTG	cis-acting regulatory element involved in light responsiveness	48

ACE	AAAACGTTTA	cis-acting element involved in light responsiveness	32
ATCT-motif	AATCTAATCT	part of a conserved DNA module involved in light responsiveness	31
MRE	AACCTAA	MYB binding site involved in light responsiveness	28
AE-box	AGAAACAA	part of a module for light response	27
as-2-box	GATAatGATG	involved in shoot-specific expression and light responsiveness	22
AT1-motif	ATTAATTTTACA	part of a light responsive module	21
ATC-motif	AGTAATCT	part of a conserved DNA module involved in light responsiveness	9
ACA-motif	ATCGACAGCCATT	part of gapA in (gapA-CMA1) involved with light responsiveness	3
ATCC-motif	CAATCCTC	part of a conserved DNA module involved in light responsiveness	1
CAG-motif	GAAAGGCAGAC	part of a light response element	1
C-box	ACGAGCACCGCC	cis-acting regulatory element involved in light responsiveness	1
W box	TTGACC	WRKY binding site involved in abiotic stress responsiveness	35
TC-rich repeats	GTTTTCTTAC	cis-acting element involved in defense and stress responsiveness	47
HSE	AAAAAATTTC	cis-acting element involved in heat stress responsiveness	55
LTR	CCGAAA	cis-acting element involved in low-temperature responsiveness	26
TCA-element	GAGAAGAATA/CCATCTTTTT	cis-acting element involved in salicylic acid responsiveness	41
ABRE	GCAACGTGTC	cis-acting element involved in the abscisic acid responsiveness	37
CGTCA-motif	CGTCA	cis-acting regulatory element involved in the MeJA-responsiveness	44
TGACG-motif	TGACG	cis-acting regulatory element involved in the MeJA-responsiveness	44
ERE	ATTTCAAA	ethylene-responsive element	26
GARE-motif	AAACAGA	gibberellin-responsive element	28
P-box	CCTTTTG	gibberellin-responsive element	25
MBS	TAACTG	MYB binding site involved in drought-inducibility	50
TATC-box	TATCCCA	cis-acting element involved in gibberellin-responsiveness	16
AuxRR-core	GGTCCAT	cis-acting regulatory element involved in auxin responsiveness	10
WUN-motif	TCATTACGAA	wound-responsive element	16
TGA-element	AACGAC	auxin-responsive element	15
5UTR Py-rich stretch	TTTCTTCTCT	cis-acting element conferring high transcription levels	36
A-box	CCGTCC	cis-acting regulatory element	14
ARE	TGGTTT	cis-acting regulatory element essential for the anaerobic induction	56
ATGCAAAT motif	ATACAAAT	cis-acting regulatory element associated to the TGAGTCA motif	26
AT-rich element	ATAGAAATCAA	binding site of AT-rich DNA binding protein (ATBP-1)	8
AT-rich sequence	TAAAATACT	element for maximal elicitor-mediated activation (2copies)	6
Box III	CATTTACACT	protein binding site	20
Box-W1	TTGACC	fungal elicitor responsive element	35

CAT-box	GCCACT	cis-acting regulatory element related to meristem expression	23
CCAAT-box	CAACGG	MYBHv1 binding site	21
CCGTCC-box	CCGTCC	cis-acting regulatory element related to meristem specific activation	11
circadian	CAANNNNATC	cis-acting regulatory element involved in circadian control	50
EIRE	TTCGACC	elicitor-responsive element	9
ELI-box3	AAACCAATT	elicitor-responsive element	3
GC-motif	GCCCCGG	enhancer-like element involved in anoxic specific inducibility	7
GCN4_motif	CAAGCCA	cis-regulatory element involved in endosperm expression	33
MBSI	TTTTTACGGTTA	MYB binding site involved in flavonoid biosynthetic genes regulation	5
MSA-like	TCCAACGGT	cis-acting element involved in cell cycle regulation	3
O2-site	GATGACATGA	cis-acting regulatory element involved in zein metabolism regulation	28
OBP-1 site	TACACTTTTGG	cis-acting regulatory element	5
RY-element	CATGCATG	cis-acting regulatory element involved in seed-specific regulation	3
Skn-1_motif	GTCAT	cis-acting regulatory element required for endosperm expression	57
CE1	TGCCACCGG	cis-acting element associated to ABRE, involved in ABA responsiveness	2
AACA_motif	TAACAAACTCCA	involved in endosperm-specific negative expression	2
MBSII	AAAAGTTAGTTA	MYB binding site involved in flavonoid biosynthetic genes regulation	2
HD-Zip 3	GTAAT(G/C)ATTAC	protein binding site	2
SARE	TTCGACCATCTT	cis-acting element involved in salicylic acid responsiveness	1
Box II -like sequence	TCCGTGTACCA	cis-acting regulatory element	1
as1	TGACGTCA	cis-acting regulatory element involved in the root-specific expression	1
OCT	CGCGGATC	cis-acting regulatory element related to meristem specific activation	1
motif I	gGTACGTGGCG	cis-acting regulatory element root specific	1
TA-rich region	TATATATATATATATATATATA	enhancer	1
3-AF3 binding site	CACTATCTAAC	part of a conserved DNA module array (CMA3)	1
TGA-box	TGACGTAA	part of an auxin-responsive element	1
AuxRE	TGTCTCAATAAG	part of an auxin-responsive element	1
AAGAA-motif	GAAAGAA	/	48
AC-I	CCCACCTACC	/	3
AC-II	TCAACCAACTCC	/	11
box E	ACCCATCAAG	/	4
box S	AGCCACC	/	9
CTAG-motif	ACTAGCAGAA	/	13
F-box	CTATTCTCATT	/	4
GCC box	AGCCGCC	/	2

H-box	CCTACCNNNNNNNCTNNNNA	/	2
JERE	AGACCGCC	/	2
plant_AP-2-like	CGCGCCGG	/	1
TATCCAT/C-motif	TATCCAT	/	25
TCCACCT-motif	TCCACCT	/	6
Y-box	TGTGGAGGAGCA	/	2

Supplementary Table S5. Gene name and gene ID of SPLs in *Arabidopsis*, rice and *Populus*.

Gene name*	Gene ID	Gene name*	Gene ID	Gene name*	Gene ID
AtSPL1	At2g47070	OsSPL1	Os01g18850	PtSPL1	Potri.010G154000
AtSPL2	At5g43270	OsSPL2	Os01g69830	PtSPL2	Potri.002G002400
AtSPL3	At2g33810	OsSPL3	Os02g04680	PtSPL3	Potri.010G026200
AtSPL4	At1g53160	OsSPL4	Os02g07780	PtSPL4	Potri.008G197000
AtSPL5	At3g15270	OsSPL5	Os02g08070	PtSPL5	Potri.008G098600
AtSPL6	At1g69170	OsSPL6	Os03g61760	PtSPL6	Potri.014G114300
AtSPL7	At5g18830	OsSPL7	Os04g46580	PtSPL7	Potri.002G188700
AtSPL8	At1g02065	OsSPL8	Os04g56170	PtSPL8	Potri.002G142400
AtSPL9	At2g42200	OsSPL9	Os05g33810	PtSPL9	Potri.005G258700
AtSPL10	At1g27370	OsSPL10	Os06g44860	PtSPL11	Potri.003G172600b
AtSPL11	At1g27360	OsSPL11	Os06g45310	PtSPL12	Potri.008G097900
AtSPL12	At3g60030	OsSPL12	Os06g49010	PtSPL13	Potri.010G154300
AtSPL13A	At5g50570	OsSPL13	Os07g32170	PtSPL14	Potri.015G098900
AtSPL13B	At5g50670	OsSPL14	Os08g39890	PtSPL15	Potri.012G100700
AtSPL14	At1g20980	OsSPL15	Os08g40260	PtSPL16	Potri.011G055900
AtSPL15	At3g57920	OsSPL16	Os08g41940	PtSPL17	Potri.016G048500c
AtSPL16	At1g76580	OsSPL17	Os09g31438	PtSPL18	Potri.001G058600
		OsSPL18	Os09g32944	PtSPL19	Potri.001G055900
				PtSPL20	Potri.001G398200
				PtSPL21	Potri.002G142200
				PtSPL22	Potri.003G169400
				PtSPL23	Potri.004G046700
				PtSPL24	Potri.007G138800
				PtSPL25	Potri.011G116800d
				PtSPL26	Potri.014G057700
				PtSPL27	Potri.014G057800
				PtSPL28	Potri.015G060400
				PtSPL29	Potri.018G149900
				PtSPL30	Potri.017G012900
				PtSPL31	Potri.019G073600

***At, Os and Pt indicated *A. thaliana, O. sativa* and *P. trichocarpa*, respectively.**

Supplementary Figure 1. The exon/intron distribution of 14 *AtSPL* genes.

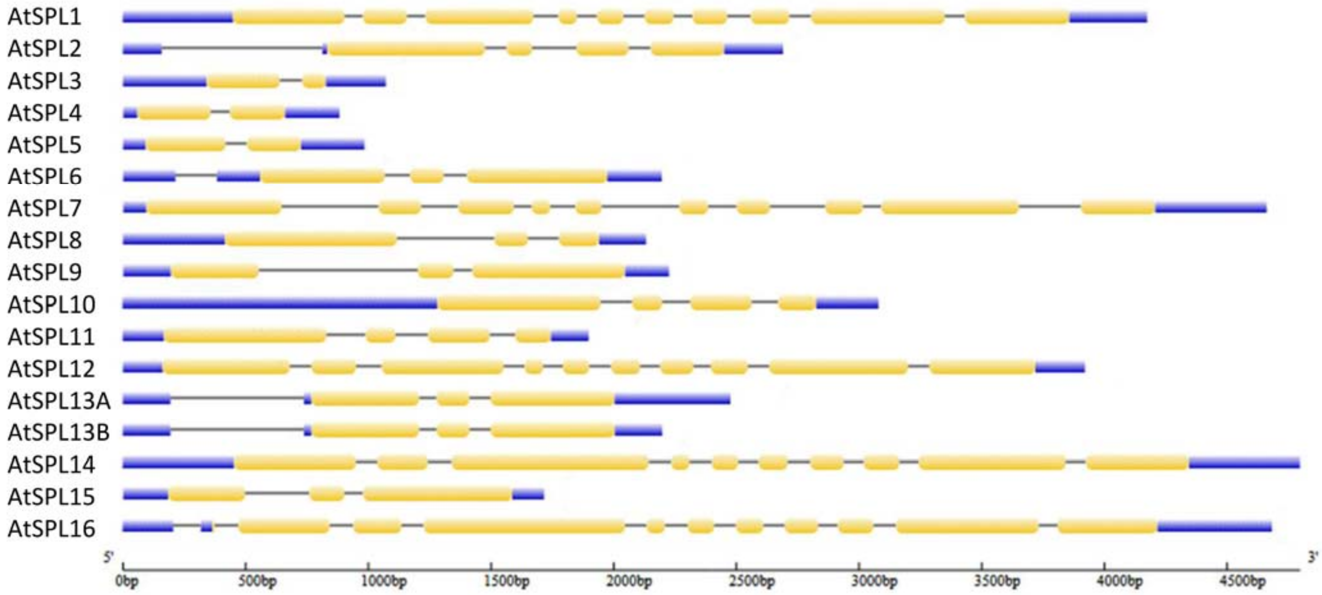

Supplementary Figure 2. Motif Logos of 20 conserved motifs of GhSPL proteins.

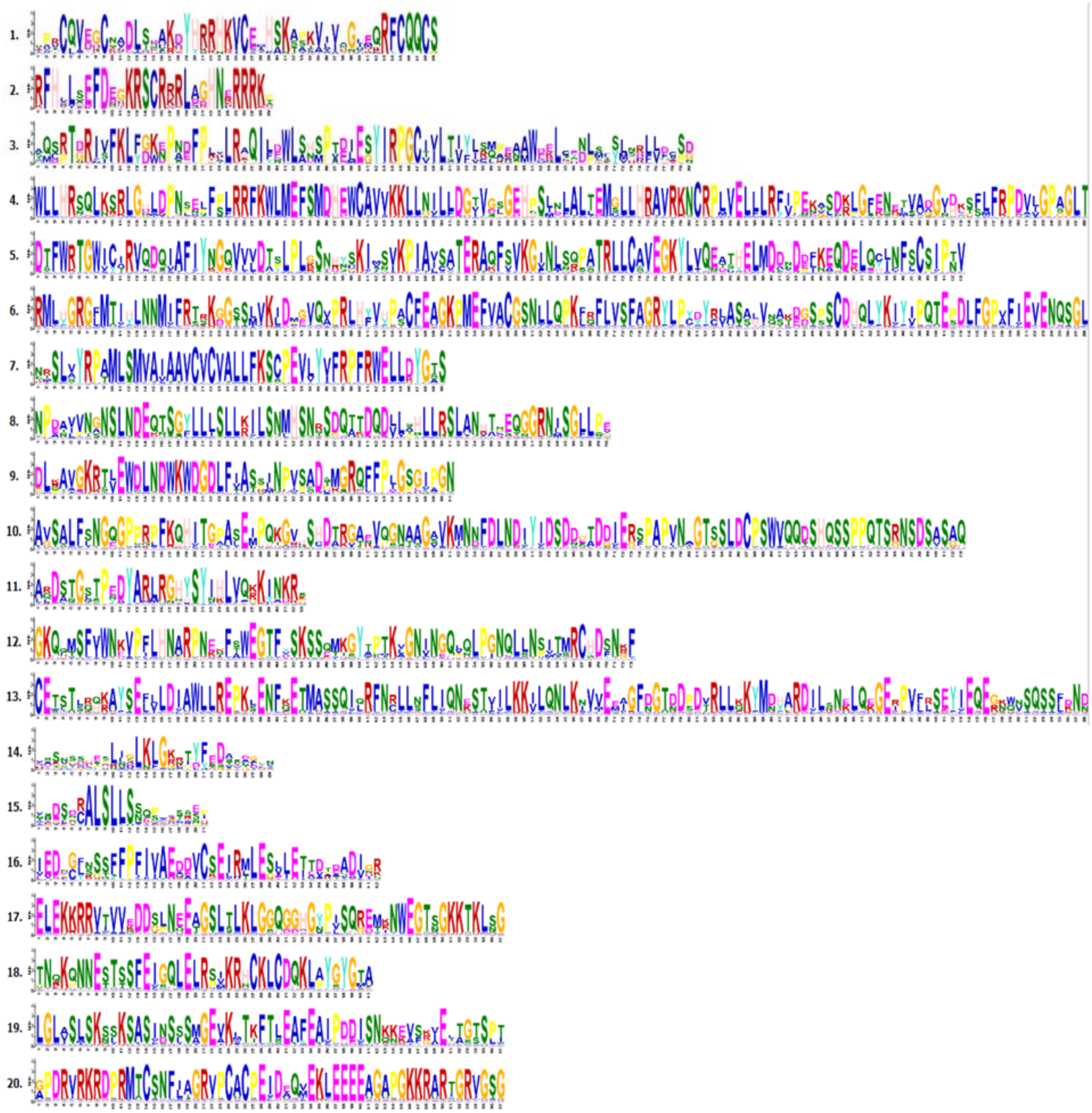

Supplement: Supplementary file 1 — Supplementary materials [file 41598_2017_18673_MOESM1_ESM.pdf]
